# ELISPOT as a Functional for Biomarker Study in Cancer Immunotherapy: Applications and Future Directions

**DOI:** 10.3390/ijms27094056

**Published:** 2026-04-30

**Authors:** Laura R. Fernández Castro, Matias Regiart, Francisco Gabriel Ortega-Sánchez, Rodrigo Rodríguez, Gonzalo Tortella, Martín A. Fernández-Baldo

**Affiliations:** 1 GENYO, Centre for Genomics and Oncological Research, Pfizer/University of Granada/Andalusian Regional Government PTS, Avenida de la Ilustración, 114, 18016 Granada, Spain; laura.fernandez@genyo.es (L.R.F.C.); gabriel.ortega@genyo.es (F.G.O.-S.); 2 IBS Granada, Instituto de Investigación Biosanitaria ibs.GRANADA, Avenida de Madrid 15, 18012 Granada, Spain; 3 Unidad de Neumología, Hospital Universitario Virgen de la Nieves, Avenida de las Fuerzas Armadas, 2, 18014 Granada, Spain; 4 Departamento de Química, Instituto de Química San Luis (INQUISAL), Universidad Nacional de San Luis, CONICET, Ejército de Los Andes 950, San Luis D5700BWS, Argentina; regiart@unsl.edu.ar; 5 Facultad de Ingeniería, Instituto de Ciencias Aplicadas, Universidad Autónoma de Chile, Temuco 4810101, Chile; 6 Centro de Excelencia en Investigación Biotecnológica Aplicada al Medio Ambiente (CIBAMA), Facultad de Ingeniería y Ciencias, Universidad de La Frontera, Av. Francisco Salazar 01145, Temuco 4811230, Chile

**Keywords:** enzyme-linked immunospot assay (ELISPOT), cancer research, biomarkers, immunotherapy

## Abstract

The Enzyme-Linked ImmunoSpot (ELISPOT) assay is a highly sensitive and widely used technique for assessing antigen-specific cellular immune responses in cancer research. By enabling the quantification of cytokine secretion at the single-cell level, particularly interferon gamma (IFN-γ), ELISPOT provides a functional readout of T cell activity with applications in both preclinical and clinical settings. This systematic review presents a structured qualitative synthesis of 78 studies investigating the use of ELISPOT in cancer immunotherapy, including cancer vaccines, oncolytic viruses, cellular therapies, immune checkpoint inhibitors, and biomarker development. Studies were selected following PRISMA guidelines from PubMed, Scopus, and Embase, focusing on both clinical and preclinical research with translational relevance. The evidence indicates that ELISPOT is widely used to validate tumor-associated antigens and neoantigens, monitor antigen-specific T cell responses during cancer immunotherapy, including immune checkpoint blockade, cancer vaccines, and adoptive cell therapies, and characterize antigen-specific T cell function. However, only a limited subset of studies establishes direct associations between ELISPOT responses and clinically meaningful outcomes. In addition, substantial variability in assay protocols and reporting criteria limits cross-study comparability and reproducibility. Overall, ELISPOT remains a valuable tool for immune monitoring in cancer research. Still, its implementation as a clinically validated biomarker requires further standardization, prospective validation, and integration with complementary analytical approaches.

## 1. Introduction

### 1.1. Cancer: Mortality Rate, Prevention, Diagnosis, and Treatment

“Cancer” is not a single clinical entity but a heterogeneous group of diseases categorized by their tissue of origin, such as the lungs, bladder, or pancreas. Despite this diversity, all cancers share a fundamental hallmark: they arise from the uncontrolled proliferation of cells that have bypassed the body regulatory checkpoints [1,2]. Globally, cancer remains a critical public health challenge, ranking as the second leading cause of death. Projections suggest that cancer-related mortality will rise sharply in the coming decades, with the vast majority of deaths resulting from metastasis, the systemic dissemination of malignant cells from a primary tumor to distant organs [3]. While primary tumors exhibit specific survival and proliferative advantages often driven by genetic mutations, the specific genetic determinants of metastatic potential remain elusive. To date, no definitive mutational profile has been identified that reliably predicts metastasis [4]. This suggests that non-mutational changes, such as alterations in three-dimensional genome organization, play a pivotal role in disease progression. By integrating genetic data with 3D genome architecture and the dysregulation of oncogenes and tumor suppressors, researchers are beginning to uncover the mechanisms governing both tumorigenesis and metastatic transition [4,5].

Improving patient outcomes relies heavily on the early detection and treatment of precancerous lesions. However, the biological transitions from healthy tissue to malignancy remain poorly understood. This knowledge gap is largely due to the scarcity of high-quality early-stage clinical samples and a lack of models that faithfully replicate human tumor development [6]. To address this, researchers have adopted sophisticated model systems, such as autochthonous mouse models, organoids, and stem cell-derived cultures, to facilitate longitudinal studies of tumor initiation [6]. When paired with high-resolution imaging, spatial multi-omics, and artificial intelligence, these models provide a comprehensive view of malignant transformation. Such integrative approaches are essential for identifying early-stage biomarkers and developing robust strategies for cancer prevention [2,7].

### 1.2. The Immune System and Types of Immune Responses

The immune system is a sophisticated network of cells, proteins, and molecular pathways that safeguards the organism against pathogens while maintaining physiological homeostasis. It comprises two synergistic branches: innate and adaptive immunity. Innate immunity serves as the primary line of defense, encompassing physical, chemical, and biological barriers, as well as specialized cellular components and soluble molecules [8]. Upon pathogen detection, leukocytes, predominantly macrophages and neutrophils, identify foreign entities, triggering an inflammatory cascade and cytokine production [8,9].

In contrast, the adaptive immune response, mediated by T and B lymphocytes, provides highly specialized cellular and humoral defenses. This system maintains a rigorous balance between self-tolerance and autoimmunity; its dysregulation can result in persistent infections or diverse immunopathologies [10,11]. A defining characteristic of adaptive immunity is its antigenic specificity. Through complex genetic rearrangements of antigen receptors during development, immature lymphocytes generate an expansive repertoire capable of recognizing virtually any non-self-molecule [12]. Following the clearance of a pathogen, most effector lymphocytes undergo apoptosis. However, a resilient population of pathogen-specific memory cells persists. These cells facilitate a rapid and robust recall response upon secondary exposure, efficiently neutralizing the threat [13].

The adaptive immune system employs diverse genetic mechanisms to generate somatic variants of antigen receptors, which are refined over time by evolutionary pressures from the environment and pathogens. While it was previously thought that adaptive immunity arose through entirely novel molecular structures, contemporary research suggests many of these “innovations” resulted from the co-option and modification of pre-existing biological systems [14]. Comparative studies across species continue to provide insights into how this specialized system diversified throughout evolutionary history.

Malignant cells produce two distinct categories of antigenic proteins recognized by the adaptive immune system. The first group, Tumor-Associated Antigens (TAAs), is typically endogenous proteins aberrantly expressed in cancer—either in ectopic tissues or at inappropriate developmental stages. This group includes differentiation antigens, cancer/testis antigens, overexpressed proteins, and occasionally, oncogenic viral proteins [15]. The second category consists of neoantigens, which arise from somatic mutations that alter protein sequences, creating novel peptides to which the immune system has no prior exposure [16,17].

For a successful immune response, these neoantigens must be processed and presented by Antigen-Presenting Cells (APCs). When immunogenic signaling is sufficient, dendritic cells capture these antigens and migrate to regional lymph nodes. There, they prime cytotoxic CD8^+^ T lymphocytes and CD4^+^ helper T cells [18,19], triggering the clonal expansion and trafficking of tumor-specific lymphocytes back to the malignancy. However, tumor microenvironments often disrupt this process, fostering immunosuppressive conditions. Immature dendritic cells and myeloid-derived populations may secrete inhibitory cytokines, recruiting regulatory T cells (Tregs). These cells dampen innate and adaptive effector functions, allowing the tumor to evade destruction [15].

Because many tumor antigens are modified self-proteins, immune tolerance remains a significant hurdle. One therapeutic strategy to circumvent this involves the use of altered homologs or xenogeneic antigens (proteins from different species). Immunization with conserved TAA paralogs has been shown to enhance the immunogenicity of otherwise “quiet” self-antigens. While breaking tolerance carries the inherent risk of inducing autoimmunity [20,21], current research aims to identify molecular patterns that selectively trigger anti-tumor immunity while minimizing autoreactive damage. This balance is central to the rational design of next-generation cancer vaccines [20].

### 1.3. Humoral and Cellular Immunity

Adaptive immunity is categorized into two primary branches: humoral and cellular immunity. These arms function synergistically to mount precise and effective responses against pathogens, including bacteria, viruses, and toxins. Although both lineages originate from a common lymphoid precursor and interact extensively during an immune challenge, each branch employs distinct functional mechanisms. Elucidating their differences and points of convergence is essential to understanding how the adaptive immune system provides comprehensive protection against a diverse array of biological threats [22].

Recent studies highlight the critical role of humoral immunity in modulating tumor progression. B cells and their secreted antibodies offer significant therapeutic potential; for instance, research has demonstrated that dimeric IgA antibodies can be delivered into malignant cells to neutralize oncogenic proteins within endosomes and facilitate their elimination from the cytosol. This discovery paves the way for the development of engineered antibodies that can target intracellular proteins previously considered undruggable. Furthermore, investigation of antibody production within intratumoral germinal centers, along with studies of various immunoglobulin classes, underscores the profound influence of the humoral response on cancer outcomes [23,24].

However, the adaptive immune system also plays a dual role in tumorigenesis. Adaptive resistance remains a formidable obstacle to an effective anti-tumor response. In this process, cancer cells actively remodel their phenotype in response to proinflammatory or cytotoxic stimuli, primarily driven by T-cell recognition. While this interaction typically triggers the release of activating cytokines intended to amplify the immune response, tumors exploit these regulatory pathways to shield themselves from T-cell-mediated destruction. By co-opting mechanisms that limit immune activity, tumors foster a permissive microenvironment that supports survival and proliferation. This dynamic resistance is the primary target of immune checkpoint inhibitors, such as PD-1 or PD-L1 blockers, which aim to disrupt the suppressive signals that T cells receive [21,25]. Overcoming this resistance is vital not only to enhance the efficacy of current inhibitors but also to guide the development of next-generation immunotherapies [26].

The adaptive immune system relies on T and B lymphocytes that express highly specific antigen receptors generated through somatic recombination. Solid tumors, however, are complex ecosystems comprising not only malignant cells but also stromal fibroblasts and a variety of immune cells, each influencing tumor behavior in distinct ways. While immune cells can promote or suppress growth, tumor cells frequently manipulate these functions to evade detection. This interaction begins at the earliest stages of oncogenesis; rapid cellular proliferation generates local stress and triggers the secretion of chemokines, such as vascular endothelial growth factor (VEGF) or members of the CXC family, to stimulate angiogenesis [27]. These signals attract immature myeloid cells of the innate immune system, which, in the absence of potent immunogenic stimuli, often trigger a “wound-healing” response that inadvertently supports the tumor [15].

Conversely, when sufficient immunogenic signals are present, these myeloid cells can process and present antigens to the adaptive immune system. This presentation typically occurs in the lymph nodes, where cytotoxic T lymphocytes and helper T cells undergo activation and clonal expansion before trafficking to the tumor site. While the adaptive immune response is fundamental to eliminating malignant cells, the clinical outcome is ultimately determined by the balance between these effector cells and the suppressive strategies within the tumor microenvironment [15].

### 1.4. ELISPOT: Cellular Immunity and Oncological Diagnosis

To accurately determine the role of adaptive immunity in oncology, highly sensitive techniques are required to assess antigen-specific immune responses. One such method is ELISPOT (Enzyme-Linked Immunospot), a platform that enables the detection of cytokine-secreting cells at single-cell resolution [28]. While widely used in clinical settings to diagnose infectious diseases such as tuberculosis or sarcoidosis, primarily because it can distinguish between latent and active infections, ELISPOT is also widely used in cancer immunotherapy research [29,30,31].

Its capacity to quantify T-cell responses against tumor-associated antigens (TAAs) makes ELISPOT particularly valuable for evaluating the efficacy of immunotherapeutic interventions. By measuring protein secretion in response to specific antigens, the assay facilitates high-precision analysis of cellular activation. This utility is critical for the development of cancer vaccines, the longitudinal monitoring of therapeutic efficacy, and the identification of novel biomarkers for diagnosis and prognosis [29].

Although ELISPOT shares structural similarities with ELISA, notably the use of enzyme-linked antibodies for detection, it differs fundamentally by providing spatial resolution at the level of individual secreting cells [28,32]. In a standard ELISPOT assay, analytes secreted by immune cells are captured by specific antibodies pre-coated onto a PVDF membrane [28,32]. These captured molecules form localized antigen–antibody complexes, which are subsequently visualized as discrete “spots” through enzymatic precipitation [33].

**Figure 1:**
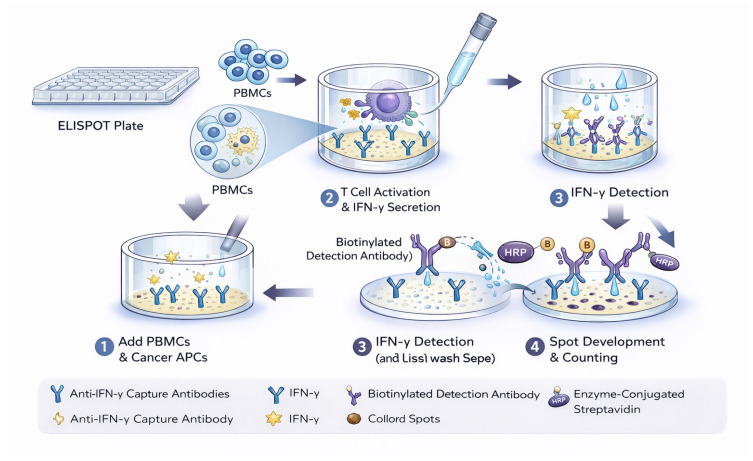
Schematic representation of the ELISPOT assay. Peripheral blood mononuclear cells (PBMCs) are seeded onto plates pre-coated with capture antibodies specific for cytokines (e.g., IFN-γ). Upon antigen-specific stimulation by antigen-presenting cells (APCs), activated T lymphocytes secrete cytokines that are captured locally by the immobilized antibodies. After incubation, biotinylated detection antibodies and enzyme-conjugated streptavidin are added, followed by a substrate that generates visible spots. Each spot corresponds to a single cytokine-secreting cell, enabling the quantification of antigen-specific cellular immune responses at the single-cell level.

Each spot corresponds to the activity of a single secreting cell [28,32], offering data on both the frequency of the responding population and the functional capacity of individual cells. Originally developed to detect antibody-secreting B cells, the technique has been widely adapted to measure T-cell responses across oncology, infectious disease, autoimmunity, and allergy research. The “one spot per cell” principle makes this method exceptionally well-suited for detecting rare immune cell populations with high specificity. In a research context, this sensitivity allows for the precise evaluation of tumor-reactive T lymphocytes and informs the rational design of targeted immunotherapies [33]. Figure 1 shows a schematic representation of the ELISPOT assay.

### 1.5. The ELISPOT Technique in Vaccine Monitoring

Cancer vaccines aim to elicit potent immune responses against tumor-associated antigens (TAAs) [34]. They are currently being investigated in various formats, including synthetic peptides, tumor lysates, whole cells, and autologous antigen-presenting cells (APCs), such as dendritic cells. In the context of melanoma, the identification of numerous immunogenic antigens has facilitated the use of standardized vaccines across patient cohorts, allowing for the assessment of anti-tumor immunity even in the absence of patient-specific neoantigens. While a primary goal of vaccine research is the discovery of reliable biomarkers to predict clinical outcomes, the fundamental immunological objective remains the induction or amplification of tumor-specific immune responses. Consequently, the use of sensitive, reproducible monitoring platforms is essential for evaluating therapeutic success [30,35].

The ELISPOT assay has emerged as a cornerstone tool for evaluating the cellular immunity elicited by these vaccines, particularly by quantifying antigen-specific cytokine-producing T cells [32]. The assay format utilizes a specialized microplate that enables the direct enumeration of individual responding cells, such as IFN-γ-secreting CD8^+^ T lymphocytes, derived from peripheral blood samples. The versatility of this technique allows it to be adapted to various antigen-presenting systems and cytokine readouts, making it highly compatible with diverse vaccination strategies [30,35].

The ability of ELISPOT to detect tumor-specific T cells present at exceptionally low frequencies makes it particularly well-suited for clinical trials. Compared to traditional methodologies, such as the chromium release assay, ELISPOT offers superior sensitivity, higher throughput, and greater ease of use. To ensure data consistency and inter-study comparability, rigorous quality assurance programs have been established to standardize assay performance across international laboratories [32,36]. These collaborative efforts in optimization and standardization facilitate the seamless integration of ELISPOT into clinical monitoring, ultimately supporting the development of effective immunotherapies designed to mobilize the immune system against malignancy [30,35]. However, ELISPOT is subject to some technical limitations and appreciable experimental variability [30,35].

This review aims to provide a comprehensive and integrative overview of ELISPOT applications in cancer immunotherapy through a structured, qualitative synthesis of the available literature, rather than a strictly quantitative systematic review. To our knowledge, no previous systematic review has comprehensively integrated the role of ELISPOT across the five major research lines in cancer immunotherapy, cancer vaccines, oncolytic viruses, cellular therapy, immune checkpoint inhibitors, and biomarkers, within a unified framework covering the 2020–2025 literature. While prior work has addressed ELISPOT from a methodological perspective [30,33,34,35] or in specific therapeutic contexts [29], the present review fills this gap by providing a structured, up-to-date synthesis of ELISPOT applications in translational oncology. Beyond bibliographic mapping, this work aims to identify emerging patterns, highlight limitations, and inform the rational development of next-generation ELISPOT-based platforms for clinical use.

## 2. Methods

This study was designed as a systematic review with a qualitative synthesis approach, following PRISMA guidelines.

### 2.1. Literature Search Strategy

A systematic literature search was conducted in three major scientific databases: PubMed, Scopus, and Embase, following the reporting guidelines of the Preferred Reporting Items for Systematic Reviews and Meta-Analyses (PRISMA) [37]. The search was completed in July 2025 and included studies published within the previous five years (2020–2025). This time frame was selected to capture the most recent developments in cancer immunotherapy. This field has undergone rapid evolution in recent years, particularly with the emergence of immune checkpoint inhibitors, cellular therapies, and personalized cancer vaccine strategies. Therefore, the focus of this review is on contemporary and translationally relevant applications of ELISPOT rather than a historical overview of the technique.

The search algorithm applied across all three databases was: (Cancer OR Oncology) AND (ELISPOT) NOT Review. This intentionally broad strategy was adopted because “ELISPOT” is a highly specific technical term with limited synonyms in the biomedical literature, thereby minimizing the risk of missing relevant studies while excluding review articles from the primary search. In PubMed, the term “ELISPOT” corresponds to the Medical Subject Heading (MeSH) descriptor “Enzyme-Linked Immunospot Assay”, which was implicitly captured by the keyword search. In Scopus and Embase, equivalent controlled vocabulary terms (Emtree: “enzyme-linked immunospot assay”) were similarly retrieved via keyword search. The complete search strings for each database were as follows:

PubMed: (Cancer OR Oncology) AND (ELISPOT) NOT Review
Scopus: (Cancer OR Oncology) AND (ELISPOT) AND NOT (DOCTYPE, re)
Embase: (Cancer OR Oncology) AND (ELISPOT) AND NOT [review]/lim

No database-specific indexing (e.g., MEDLINE), language, geographic origin, or publication type restrictions beyond those specified in the inclusion criteria were applied at the database level. Duplicate records were removed across databases during the screening process. To ensure methodological transparency and compliance with reporting standards, this review was conducted and reported in accordance with PRISMA 2020 guidelines. A detailed PRISMA checklist is provided in Supplementary Materials Table S1.

### 2.2. Study Selection and Screening Process

The study selection process followed PRISMA guidelines. Two independent reviewers performed the screening in three sequential stages: title, abstract, and full-text evaluation. Discrepancies regarding study inclusion were resolved through discussion and consensus. When necessary, a third reviewer was consulted to reach a final decision. This procedure was adopted to improve the transparency, consistency, and reproducibility of the study selection process.

Studies were included if they met the following criteria: (i) focused on the application of ELISPOT in the investigation of any cancer; (ii) published in peer-reviewed journals; (iii) written in English; and (iv) conducted in human subjects or in preclinical models with direct translational relevance to human oncology, including murine tumor models, in vitro cellular systems, and ex vivo assays.

Studies were excluded if they met any of the following criteria: (i) addressed infectious diseases without oncological relevance; (ii) focused exclusively on the technical development of ELISPOT readout methods without application to cancer; (iii) were book chapters, conference abstracts, or grey literature; or (iv) were review articles.

### 2.3. Data Synthesis and Analysis

Due to the heterogeneity in study designs, cancer types, immunotherapeutic approaches, and ELISPOT assay protocols, a quantitative meta-analysis was not feasible. Therefore, the results were qualitatively synthesized to identify consistent patterns, methodological trends, and current research gaps. Additionally, variability in ELISPOT assay protocols, including antigen selection, cell preparation, and readout criteria, contributes to inter-study heterogeneity. This qualitative synthesis and classification approach was used to facilitate cross-study comparison and to support the integrative analysis presented in the Section 3.

### 2.4. Limitations

A formal risk of bias assessment was not conducted, as the primary objective of this review was to provide a comprehensive and integrative overview of ELISPOT applications in cancer immunotherapy rather than to perform a quantitative synthesis. Nevertheless, this may represent a limitation and should be considered when interpreting the findings. The methodological variability across ELISPOT assays further limited the applicability of standardized bias assessment tools. Furthermore, this review was not prospectively registered in PROSPERO or any equivalent systematic review registry, which is acknowledged as an additional methodological limitation.

Additionally, a structured qualitative assessment of study rigor was performed based on ELISPOT standardization level, reporting quality, and clinical outcome association (Table S2).

### 2.5. Organization of Information

A Google Drive spreadsheet was created to systematically and consistently collect relevant information from each study. The following variables were considered:

- DOI;
- Title;
- First author;
- Institution;
- Year of publication;
- Type of study (clinical trial, observational study, etc.);
- Application of ELISPOT (vaccine response monitoring, cellular therapy, etc.);
- Relevance of the findings (brief qualitative summary);
- Presence or absence of correlation with clinical endpoints (overall survival, tumor burden, treatment response).

This approach enabled classification of articles into groups based on the type of study conducted, thereby facilitating comparative analysis.

Selected figures are included to illustrate key experimental workflows and conceptual aspects of ELISPOT applications. These comprise original schematics created by the authors, as well as adapted or reproduced figures from published studies, with appropriate attribution where required.

## 3. Results and Discussion

### 3.1. Literature Search

A total of 240 articles were identified in PubMed, 267 in Scopus, and 834 in Embase, yielding a final total of 1341 studies. A total of 310 duplicate articles were identified and removed using the tools available in Google Drive. The remaining 1031 studies were manually screened in two phases. First, during the screening phase, titles and abstracts were analyzed to identify studies that explicitly mentioned both cancer and ELISPOT. Articles that did not meet these inclusion criteria were excluded, reducing the number of eligible studies to 81.

These 81 articles were retrieved, and their full texts were reviewed. In a final eligibility phase, three additional studies were excluded: one addressing infections [38], one focused on the development of ELISPOT readout methods [39], and one that was a book chapter [29]. Consequently, 78 studies were included in the final analysis.

### 3.2. General Description of the Selected Articles

**Figure 2:**
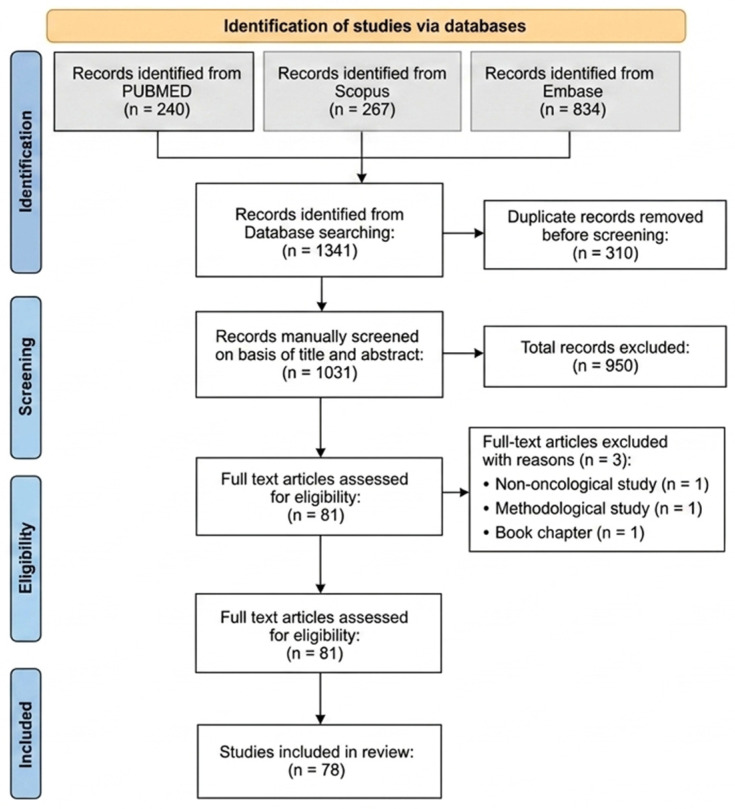
Flow diagram of article selection for this review.

Each article included in this review uses the ELISPOT assay to evaluate T cell-mediated responses in oncological research. These studies span 2020 to 2025 and cover a wide range of cancer types, immunotherapeutic strategies, and experimental designs. Figure 2 shows a flow diagram of article selection for this review.

For clarity, the studies were organized into five research lines: (i) cancer vaccines, (ii) biomarkers, (iii) cellular therapy, (iv) oncolytic viruses, and (v) immune checkpoint inhibitors. This classification enables a more structured exploration of ELISPOT’s contributions across different immuno-oncology approaches.

The research line focused on cancer vaccines includes 44 articles published between 2020 and 2025. This is the largest group in the present review and primarily focuses on measuring CD8^+^ and CD4^+^ T lymphocyte responses to determine IFN-γ production. This way, researchers assess the functionality of adaptive immunity when stimulated with different peptides, neoantigens, tumor-associated antigens, and related targets.

The section on oncolytic viruses comprises 7 studies published between 2021 and 2025. In these works, ELISPOT is highlighted as a crucial tool for demonstrating the induction of tumor-specific or systemic T cell responses following viral administration. Several studies combine virotherapy with immune checkpoint inhibitors, thereby amplifying the immunogenic effects detected by ELISPOT.

Regarding cellular therapy, seven studies published between 2020 and 2025 were included in this category. This group of articles emphasizes the role of ELISPOT in immunological characterization before and after the direct administration of modified or selected live cells to patients, primarily through therapies such as genetically engineered T cell receptor-modified lymphocytes (TCR-T) or dendritic cell-based vaccines.

The section on immunotherapy and immune checkpoint inhibitors comprises 14 papers, making it the second-largest category in this review and encompassing studies published between 2020 and 2025. This type of immunotherapy primarily focuses on analyzing mechanisms of immune system inhibition to exploit them for cancer treatment.

Finally, the research line addressing biomarkers includes six studies published in 2022, 2024, and 2025. This section considers ELISPOT as a tool for identifying potential biomarkers useful for cancer detection, diagnosis, and treatment.

**Figure 3:**
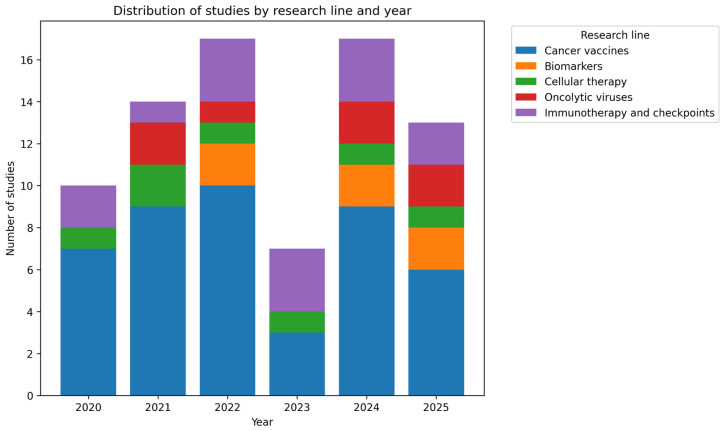
Distribution of studies by research line and year.

Taken together, all these studies support the utility of ELISPOT in evaluating cell-mediated immune responses during tumor development. The assays predominantly focus on IFN-γ analysis to measure lymphocyte activity, providing valuable information that can be leveraged to develop vaccines, immunotherapies, oncolytic virus strategies, and related approaches. Figure 3 shows a distribution of studies by research line and year.

While this classification facilitates structured presentation, interpreting results requires cross-study comparisons and critical evaluation of methodological variability and translational relevance, as discussed in the following sections.

### 3.3. Research Lines

The studies presented below employ ELISPOT to measure activity at the single-cell level and encompass assays conducted in different species, both in vitro and in vivo. These articles highlight the adaptability of ELISPOT and its importance for evaluating cellular immunity, a crucial step in understanding the molecular and cellular mechanisms of cancer. In this way, research can develop various approaches that enable earlier disease detection and support personalized treatment strategies.

#### 3.3.1. Cancer Vaccines

The primary objective of therapeutic cancer vaccines is to induce antigen-specific T cell responses directed against tumor-associated antigens and neoantigens, thereby enhancing anti-tumor immunity and improving clinical outcomes. Within this framework, ELISPOT has been consistently employed as a functional platform to validate candidate epitopes, quantify vaccine-induced immune activation, and correlate immunogenicity with therapeutic efficacy.

In breast cancer, early ELISPOT-based studies focused on determining whether T lymphocytes could effectively discriminate between self-derived and mutated antigens. Wang et al. [40] investigated HLA-A2^+^ patients and demonstrated that both wild-type (WT) and mutant (MT) peptides induced IFN-γ secretion, albeit to differing degrees. This finding was particularly relevant, as it indicated that non-mutated self-antigens may retain a measurable degree of immunogenicity, expanding the potential antigenic repertoire for vaccine strategies.

Subsequent research refined neoantigen prioritization. Mistretta et al. [41] evaluated 15 candidate neoantigens in breast cancer patients and identified ENDIKPKF as a highly immunogenic peptide, eliciting responses twice as strong as the positive control and 5 times stronger than the negative control. This study underscored the importance of functional validation in narrowing down computationally predicted neoantigens. Extending these observations to a more aggressive subtype, Zhang et al. [42] demonstrated that neoantigen-based DNA vaccines in triple-negative breast cancer induced specific T cell responses against 45 of 47 evaluated neoantigens following vaccination and in vitro culture, highlighting the feasibility of personalized vaccine approaches in clinically challenging settings.

The immunological scope was further broadened by [43], who examined antigen presentation in the context of HLA class II molecules. Their findings showed that neoantigen-derived peptides were recognized by CD4^+^ T lymphocytes, indicating that vaccine-induced anti-tumor responses may involve coordinated activation of CD8^+^ cytotoxic and CD4^+^ helper T cells. Collectively, these studies suggest that breast cancer immunogenicity extends beyond strictly MHC class I–restricted mechanisms.

More recently, ref. [44] expanded antigen discovery strategies by comparing translatome-derived peptides with conventional peptides in lung cancer models. The stronger ELISPOT responses observed against translatome-derived peptides suggest that non-canonical translation products may represent an underexplored source of immunogenic targets, reinforcing the assay’s utility in broadening antigen repertoires.

In colorectal cancer, ELISPOT has similarly been instrumental in validating the functionality of neoantigens. Corulli et al. [45] demonstrated that epitope-specific T lymphocytes could recognize recombinant proteins naturally processed by tumor cells, thereby confirming biological relevance beyond synthetic peptide stimulation. Yu et al. [46] further showed IFN-γ secretion by T lymphocytes reactive to neoantigens in patient-derived samples, indicating that immune cells could effectively target autologous tumor cells. Complementing these findings, ref. [47] compared canonical and cryptic tumor-derived peptides and identified several that could induce functional responses against patient tumors. Zhang et al. [48] subsequently correlated ELISPOT response intensity with HLA-binding affinity and CD8^+^ T-cell activation, identifying 100 µg per neoantigen as the optimal concentration for maximal functional response. These studies collectively illustrate how ELISPOT informs both antigen validation and vaccine optimization.

In ovarian cancer, the application of ELISPOT extended beyond immunogenicity assessment to prognostic evaluation. Rocconi et al. [49] reported that patients with recurrent disease treated with the Vigil vaccine who exhibited IFN-γ secretion experienced improved long-term survival, suggesting that functional cytokine production may serve as a predictive biomarker. In a complementary preclinical setting, ref. [50] demonstrated that the TSA-Nutu-R vaccine induced significantly stronger IFN-γ and IL-4 secretion in splenocytes compared with controls, reflecting activation of both Th1 and Th2 immune pathways.

Similarly, in prostate cancer, ELISPOT has been applied across multiple therapeutic contexts. Pachynski et al. [51] evaluated PA2024- and prostatic acid phosphatase (PAP)-specific T cell responses following administration of recombinant human interleukin-7 (rhIL-7). Although response frequency did not significantly change, the overall increase in lymphocyte counts suggested expansion of antigen-specific effector populations. More recently, ref. [52] assessed cancer-specific T cell responses following irreversible electroporation (IRE) and observed de novo responses or amplification of pre-existing responses against prostate and viral antigens, indicating therapy-induced immune modulation.

Beyond these tumor types, ELISPOT has also been used to evaluate immunological responses in pancreatic, brain, and cervical cancers. In murine pancreatic cancer models, ref. [53] demonstrated that combined nicotinamide (NAM) and gemcitabine (GEM) therapy reduced immunosuppressive cell populations and enhanced T cell activation. In glioblastoma, ref. [54] showed that cytotoxic T lymphocytes generated via induced pluripotent stem cell-derived dendritic cells (iPSC/DCs) produced higher IFN-γ levels upon recognition of HLA-matched tumor cells compared with conventional dendritic cell-based approaches. In cervical cancer, ref. [55] reported a ≥twofold expansion of HPV16- and HPV18-specific T cells following chemoradiotherapy and ipilimumab treatment, reflecting treatment-driven immune activation.

Numerous additional studies explored novel antigenic sources and personalized vaccine platforms. Akazawa et al. [56] demonstrated that EGFR T790M/C797S mutant peptides induced functional CTLs in HLA-A*02:01^+^ donors. Thomas et al. [57] observed IFN-γ secretion in PBMCs stimulated with LDHC-derived peptides, particularly after dendritic cell-mediated presentation. Handlos Grauslund et al. [58] identified CALRLong36-induced IFN-γ responses in patients with chronic myeloproliferative neoplasms. Gao et al. [59] reported spontaneous T cell reactivity against mutated variants, including KEAP1, KIAA0408, and related genes, findings reinforced by [60,61]. In nasopharyngeal carcinoma, ref. [62] demonstrated strong CTL responses against LMP2 that correlated with improved 5-year survival outcomes.

Technological advancements further expanded ELISPOT applications. Chen et al. [63] reported antigen-specific activation with the iNeo-Vac-P01 vaccine, while [64] demonstrated CD4^+^ T cell responses against ARG1-derived peptides. Cecil et al. [65] confirmed immunogenicity of the TSA-Nutu-R vaccine in preclinical models, and [66] validated mutated PPM1F peptides in vaccinated patients. Feola et al. [67] employed ELISPOT to select immunogenic peptides for personalized oncolytic vaccine design, whereas [68] demonstrated robust neoantigen-specific responses following vaccination.

**Figure 4:**
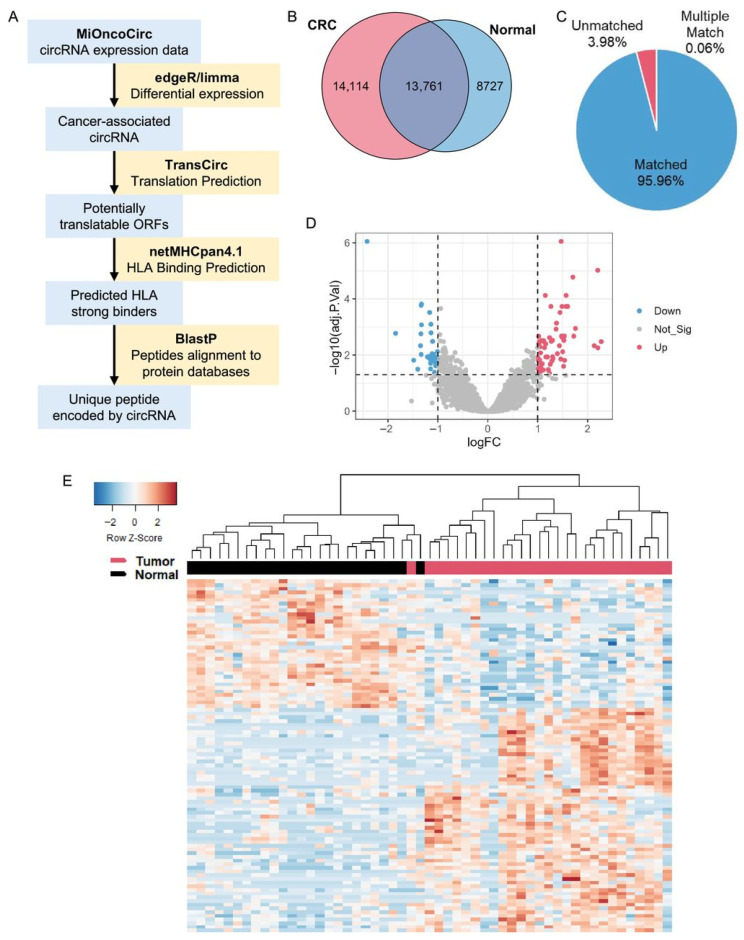
circRNA expression in MiOncoCirc CRC samples. (**A**) circRNA neoantigen prediction pipeline. circRNA expression data were acquired from MiOncoCirc, and differentially expressed circRNAs were analyzed using R package edgeR. circRNAs were then mapped to the TransCirc database for ORF and translation prediction. HLA-A*11:01 binding affinity of potentially translatable ORFs was predicted using netMHCpan4.1, and shortlisted strong binders were aligned to the NCBI protein database using BlastP to screen for novel peptides. (**B**) circRNA detected in CRC and normal samples. Only overlapped circRNAs were kept for downstream analyses (**C**) fraction of circRNA mapped to TransCirc. The genomic locations of circRNA were used as identifiers to map to the TransCirc database. Only uniquely matched circRNAs were kept. (**D**) Volcano plot of differentially expressed circRNA. (**E**) Expression of differentially expressed circRNA in tumor and normal samples. circRNA, circular RNA; CRC, colorectal cancer; HLA, human leukocyte antigen; ORF, open reading frame. Reproduced from [75] under the terms and conditions of the Creative Commons Attribution Non-Commercial (CC BY-NC) license (https://creativecommons.org/licenses/by-nc/4.0/).

Combination strategies also showed promising results. Salvatori et al. [69] reported tumor regression in murine models treated with DNA vaccines plus αCTLA-4 blockade. Maruoka et al. [70] demonstrated that dendritic cells loaded with tumor spheroids or ivtRNA generated functional CTLs capable of recognizing neoantigen peptides. Shafer et al. [71] (2024) analyzed clonotype-specific recognition of ESR1-mutant cells, demonstrating variant-dependent antigen specificity restricted by HLA-B*40:02. Further innovation included HER2-targeting DNA vaccines [72], CpG-enhanced gp100 responses [73], L-pampo™ adjuvant optimization [74], and circRNA-based neoantigen platforms [75]. Figure 4 shows a schema of circRNA expression in MiOncoCirc CRC samples.

Recent combinations and nanotechnology-based platforms continue to demonstrate enhanced immune activation. Peters et al. [76] showed that the oncolytic virus MEM-288 induced systemic immunity, leading to tumor reduction. Ishizuka et al. [77] reported increased IFN-γ and IL-2 production following combined nanoformulation and photodynamic therapy. Li et al. [78] demonstrated robust multi-neoantigen responses using the AECM@PC7A nanovaccine. Huang et al. [79] confirmed enhanced splenic IFN-γ responses with the HPPS-OVA@RMn platform, while [80] showed that OligoDOM-containing mRNA sequences induced strong antigen-specific T cell activation across multiple tumor models.

Taken together, these findings illustrate that ELISPOT has been consistently employed not only to confirm neoantigen immunogenicity but also to optimize antigen selection, refine vaccine platforms, evaluate combinatorial regimens, and establish functional correlates of clinical benefit. Across diverse tumor types and technological innovations, the assay remains central to the development and validation of personalized cancer vaccine strategies.

Taken together, these studies reveal consistent use of ELISPOT as a functional validation tool for neoantigen immunogenicity and vaccine-induced T cell activation. However, substantial variability exists in assay standardization, including antigen selection, stimulation protocols, and positivity thresholds, as reflected in Table S1. Importantly, while several studies report robust IFN-γ responses, only a limited subset demonstrates clear associations with clinical outcomes, highlighting a gap between immunological readouts and translational relevance.

#### 3.3.2. Oncolytic Viruses

Oncolytic virotherapy represents a therapeutic strategy that leverages the ability of natural or genetically engineered viruses to selectively infect and lyse tumor cells while simultaneously promoting anti-tumor immune responses. Within this context, ELISPOT has been predominantly employed as a functional assay to quantify virus-induced T cell activation, either following monotherapy or in combination with immune checkpoint inhibitors, peptide vaccination, or local tumor-directed interventions.

Early preclinical evidence demonstrated the capacity of oncolytic platforms to induce mutation-specific immunity. In a murine model, ref. [81] showed that vaccinated animals developed T cell responses against four tumor-associated mutations. Notably, co-administration of mutated peptides with adenovirus and myxoma-based oncolytic viruses (Ad + MRB) significantly enhanced antigen-specific T cell responses and improved survival in melanoma and colorectal carcinoma models compared with virus-only treatment. These findings underscored the synergistic potential of combining neoantigen targeting with viral-mediated immune stimulation.

In parallel, ref. [82] evaluated the cytotoxic activity of T lymphocytes against 4T1 breast cancer cells and demonstrated that treatment with DTK-Armed-VACV induced robust IFN-γ secretion, confirming effective activation of cytotoxic T lymphocytes (CTLs). Together, these early studies established ELISPOT as a sensitive platform for detecting virus-induced cellular immunity in preclinical models.

Subsequent investigations examined the impact of intratumoral viral administration on systemic immune activation. Ding et al. [83] analyzed splenocytes from mice treated with an oncolytic virus and identified antigen-specific T cell responses in the spleen, indicating that local viral therapy can elicit systemic immunity. Expanding on this concept, ref. [84] employed a combined ELISPOT and quantitative PCR (qPCR) approach to evaluate a conditionally replicating adenoviral vector system (CAdVEC). While initial treatment induced adenovirus-specific T cell responses, later stages revealed immune evasion by helper-dependent adenovirus (HDAd)-infected cells. This study highlighted the delicate balance between therapeutic efficacy and viral immune escape, illustrating the dynamic interplay between viral persistence and host immunity.

Further evidence of potent virus-driven immunogenicity was provided by [85], who assessed cytokine production following ex vivo antigenic stimulation in vaccinated animals. ELISPOT analysis demonstrated strong IFN-γ-producing responses against the E7 antigen, particularly in groups receiving the oncolytic adenovirus, indicating superior immunogenicity compared with DNA vaccination alone. These findings reinforce the concept that viral vectors may amplify antigen presentation and enhance cytotoxic T cell activation.

More recently, research has increasingly focused on combinatorial approaches designed to amplify immune activation. Sun et al. [86] investigated the combined effects of radiofrequency tumor ablation (iRFA), the oncolytic peptide LTX-315, and anti-CTLA-4 blockade in Hepa1-6 tumor models. ELISPOT analysis revealed a significant expansion of IFN-γ-producing CD8^+^ T lymphocytes, demonstrating that integrating physical tumor disruption, peptide-mediated oncolysis, and immune checkpoint inhibition can synergistically enhance cytotoxic immunity.

**Figure 5:**
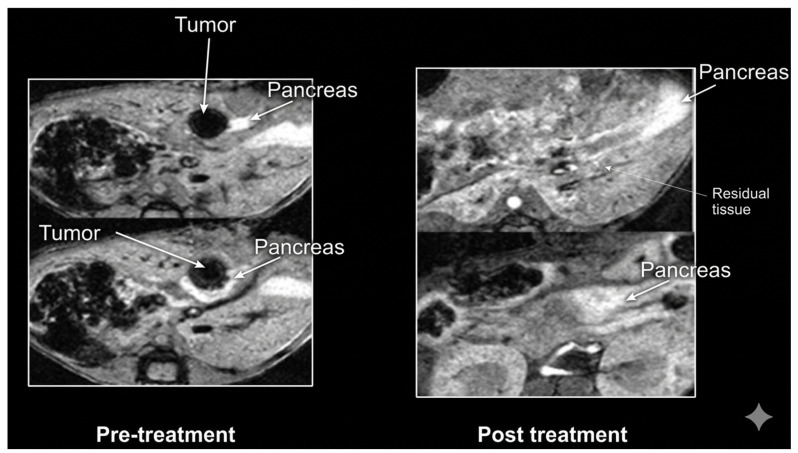
Administration of SV vectors to SCID mice bearing orthotopically growing human pancreatic tumor cells results in eradication of the tumor cells. Reproduce from [87] under the terms and conditions of the Creative Commons Attribution (CC BY) license (https://creativecommons.org/licenses/by/4.0/).

Beyond immediate effector responses, the induction of long-term immunological memory has emerged as a critical objective in oncolytic virotherapy. Opp et al. [87] evaluated combination treatment with SV.IL12 and αOX40 in a pancreatic cancer model, demonstrating that previously treated mice rechallenged with tumor cells exhibited persistent IFN-γ-producing CD4^+^ memory T cells (Figure 5). ELISPOT confirmation of memory responses indicates that such combinatorial strategies may confer durable functional immunity rather than transient activation.

Collectively, these studies illustrate a clear conceptual progression in the field. Initial work established the capacity of oncolytic viruses to induce mutation-specific T cell responses in murine models. Subsequent investigations explored systemic immune activation, addressed viral immune evasion mechanisms, and ultimately expanded toward multimodal regimens designed to enhance both effector function and memory formation. Despite challenges related to viral clearance and immune escape, the consistent detection of IFN-γ-producing lymphocytes across diverse platforms underscores the utility of ELISPOT as a robust functional tool for evaluating oncolytic virotherapy-induced cellular immunity.

Despite consistent detection of IFN-γ-producing T cells across studies, the magnitude and durability of responses vary considerably depending on viral platform, combination strategy, and experimental model. Moreover, most studies rely on preclinical systems, limiting direct clinical extrapolation. These discrepancies underscore the need for standardized ELISPOT protocols and longitudinal clinical validation.

#### 3.3.3. Cellular Therapy

Cellular immunotherapies are designed to restore or amplify the host’s capacity to mount effective anti-tumor immune responses. These approaches encompass adoptive transfer of tumor-infiltrating lymphocytes (TILs), T cell receptor-engineered lymphocytes (TCR-T), and dendritic cell-based vaccines aimed at priming or enhancing antigen-specific cytotoxic activity. Across these modalities, ELISPOT has served as a functional assay to quantify antigen-driven T cell activation and to assess therapeutic efficacy in both clinical and preclinical settings.

Clinical studies in breast cancer first illustrated the value of ELISPOT in detecting antigen-specific immune activation within patient-derived samples. Van Pul et al. [88] analyzed sentinel lymph nodes. They demonstrated that CpG stimulation, combined with a STAT3 inhibitor, enhanced T cell reactivity against mammaglobin-A (MAM-A), indicating reversal of local immunosuppression. Extending these findings, ref. [89] evaluated circulating T lymphocytes from breast cancer patients and observed significantly higher IFN-γ production following exposure to nucleolin (NCL) peptides compared with controls and non-activated monocytes. Together, these studies underscore ELISPOT’s capacity to capture clinically relevant antigen-specific activation directly within the patient’s immune compartment.

In parallel, murine models enabled evaluation of more complex combinatorial strategies integrating immunotherapy and chemotherapy. Gao et al. [90] investigated a triple therapy regimen (CpG + α-OX40 + doxorubicin) in breast cancer. They demonstrated that splenocytes from treated mice produced elevated IFN-γ levels when stimulated with mitomycin-treated tumor cells or mutated peptides. These findings highlight ELISPOT’s utility in quantifying synergistic enhancement of T cell activation following multimodal intervention.

Dendritic cell (DC)-based vaccination strategies have likewise benefited from functional validation using ELISPOT. Liao et al. [91] developed a vaccine targeting cancer stem cells (CSCs) through peptides derived from aldehyde dehydrogenase (ALDH). They showed that splenic T lymphocytes from vaccinated animals produced significantly higher IFN-γ levels in response to ALDH^high CSCs compared with ALDH^low cells. This result is particularly relevant, as CSC populations are frequently implicated in tumor recurrence and therapeutic resistance. However, not all DC-based approaches demonstrated equivalent immunogenicity. Miyaguchi et al. [92] reported that dendritic cells loaded with glioblastoma stem cell (GSC) lysates failed to induce a significant increase in IFN-γ secretion compared with controls, suggesting limited immunogenic potential of certain stem cell-derived antigen preparations. These contrasting findings illustrate the importance of functional assays in distinguishing effective from suboptimal antigen-loading strategies.

Beyond antigen presentation platforms, ELISPOT has also been instrumental in evaluating genetically engineered T cell therapies. Shirosaki et al. [93] generated TCR-T cells targeting the AP2S1 neoantigen and demonstrated that ALDH^high tumor cells evaded AP2S1-specific cytotoxic activity due to reduced HLA class I expression. This observation highlights how ELISPOT can reveal immune escape mechanisms that compromise engineered T cell efficacy, particularly in tumor subpopulations associated with resistance.

**Figure 6:**
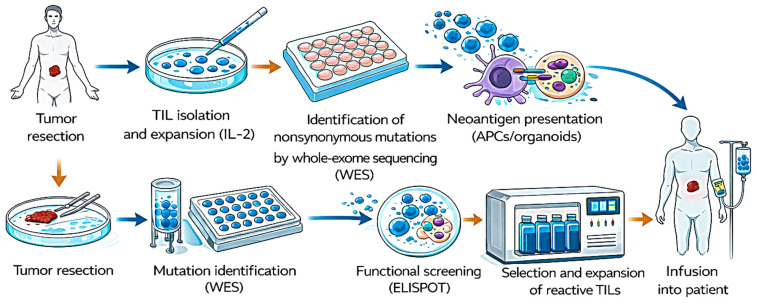
Schematic representation of tumor-infiltrating lymphocyte (TIL) expansion and neoantigen screening workflow in cancer immunotherapy. Adapted from [94] under the terms and conditions of the Creative Commons Attribution (CC BY) license.

Adoptive transfer of tumor-infiltrating lymphocytes represents another key cellular strategy in which ELISPOT has provided critical functional insight. Gustafson et al. [94] analyzed TIL cultures derived from metastatic skin cancer patients and observed substantial heterogeneity in neoantigen recognition. While some peptide fragments induced strong IFN-γ secretion, others elicited weak or absent responses. This variability underscores the complexity of intratumoral antigenic landscapes and reinforces the need for functional screening to guide epitope prioritization in personalized therapy. Figure 6 shows a schema of TIL growth and neoantigen screening in metastatic epithelial cancers.

Collectively, these studies demonstrate that ELISPOT is not merely a tool for measuring cytokine release but a platform capable of dissecting functional synergy between therapeutic modalities, identifying immune evasion pathways, and capturing heterogeneity within tumor-reactive lymphocyte populations. In the context of cellular immunotherapy, the assay provides a sensitive readout of antigen-specific effector competence. It remains central to the optimization of strategies targeting resistant tumor cell fractions and cancer stem cell-associated mechanisms.

Notably, these studies reveal significant heterogeneity in antigen recognition and functional response, particularly in patient-derived T cell populations. While ELISPOT effectively captures this variability, the lack of standardized thresholds and inconsistent reporting limits cross-study comparability and biomarker validation.

#### 3.3.4. Immunotherapy and Immune Checkpoints

The study of immune checkpoints centers on understanding the regulatory mechanisms that restrain anti-tumor immunity and on exploiting these pathways therapeutically to restore effective immune responses. Within this framework, ELISPOT has been widely employed to quantify antigen-specific cytokine secretion before and after immunomodulatory interventions, thereby enabling functional assessment of therapeutic efficacy and identification of potential immune biomarkers.

Initial investigations highlighted the impact of tumor-associated immunosuppressive cells on effector function. In murine models of oral cancer and in patients with head and neck squamous cell carcinoma, ref. [95] demonstrated that myeloid-derived suppressor cells (MDSCs) significantly reduced IFN-γ production by natural killer (NK) cells. Importantly, this suppression was partially reversible with functional inhibitors, demonstrating that ELISPOT can detect restoration of immune activity following targeted intervention. In the same year, ref. [96] employed HLA-A*02:01 humanized mice vaccinated with the HSFX1 antigen and confirmed the induction of antigen-specific T cell responses, reinforcing the utility of ELISPOT for validating antigen-driven immune activation in checkpoint-relevant contexts.

The transition to clinical studies expanded these observations into patient-based settings. In prostate cancer, ref. [97] applied ELISPOT to evaluate immune responses following recombinant human interleukin-7 (rhIL-7) administration and detected increased frequencies of antigen-specific T lymphocytes reactive to PA2024 and PAP, indicating enhanced effector expansion. Similarly, in bladder cancer, ref. [98] reported that treatment with rBCG-S.FimH augmented IFN-γ production in draining lymph nodes, suggesting local immune activation. Complementing these findings, ref. [99] investigated a prime–boost vaccination strategy using chimpanzee adenovirus Oxford 1 (ChAdOx1) for priming and modified vaccinia Ankara (MVA) for boosting. Notably, intravenous administration elicited stronger and more sustained T cell responses against prostate antigens than intramuscular delivery, highlighting the influence of route of administration on functional immune outcomes detectable by ELISPOT.

Combination checkpoint blockade strategies further demonstrated the assay’s sensitivity in capturing synergistic immune enhancement. Liu et al. [100] showed that dual blockade of KIR and PD-L1 (aKIR + aPD-L1) in cervical cancer significantly increased IFN-γ and granzyme B production in NK cells compared with either agent alone, illustrating cooperative activation of cytotoxic pathways. In parallel, ref. [101] confirmed that several predicted peptides elicited antigen-specific IFN-γ secretion after prolonged stimulation. At the same time, ref. [102] observed strong CD8^+^ T cell responses in vaccinated melanoma patients, accompanied by peripheral clonal expansion. These findings underscore the assay’s ability to validate antigen specificity and track functional T cell amplification in immunotherapy settings.

ELISPOT has also been employed to explore immune dynamics beyond direct checkpoint blockade. Pichler et al. [103] did not detect SARS-CoV-2-specific responses in bladder cancer patients treated with bacillus Calmette–Guérin (BCG), indicating that certain immunotherapies may not broadly enhance unrelated antiviral immunity. Conversely, ref. [104] demonstrated that chemoimmunotherapy in metastatic non-small-cell lung cancer patients induced dynamic modulation of TERT-specific CD4^+^ Th1 cells, including expansion, contraction, or elimination of antigen-specific populations during treatment. These results highlight ELISPOT’s ability to capture temporal fluctuations in functional immune responses.

Baseline immune competence has likewise emerged as a potential predictive biomarker. In castration-resistant prostate cancer, ref. [105] reported that higher pre-treatment lymphocyte functionality, as measured by ELISPOT before radium-223 therapy, was associated with a lower tumor burden after six treatment cycles. This observation suggests that pre-existing immune responsiveness may influence therapeutic outcome.

**Figure 7:**
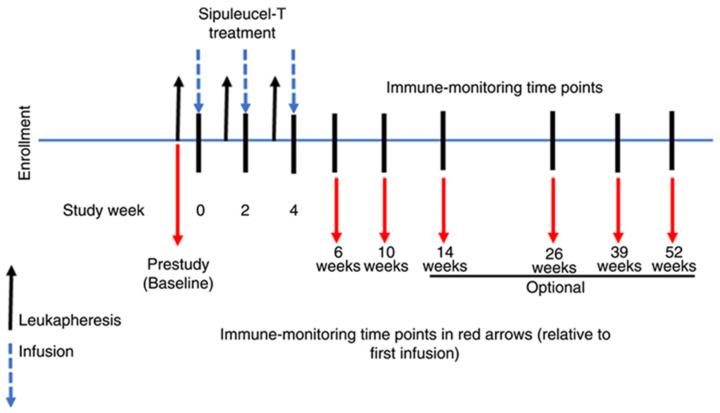
Treatment schema showing schedule of Sipuleucel-T infusions and immune evaluations. Reproduced from [107] under the terms and conditions of the Creative Commons Attribution (CC BY) license.

Additional immunomodulatory mechanisms have also been investigated. Pandey et al. [106] demonstrated that prostaglandin E2 (PGE2) increased IL-6 production in human PBMCs, illustrating how soluble mediators can reshape cytokine profiles detectable by functional assays. Heath et al. [107] showed that combination therapy with Sipuleucel-T and ipilimumab induced robust CD4^+^ and CD8^+^ T cell responses against PA2024 in prostate cancer patients, irrespective of racial background, indicating the broad applicability of combined immune-stimulation strategies. Figure 7 shows a treatment scheme of Sipuleucel-T infusions and immune evaluations.

Finally, Naik et al. [108] confirmed that tumor-infiltrating lymphocytes (TILs) from patients retained cytotoxic capacity and produced antigen-dependent IFN-γ, demonstrating preserved effector function within the tumor microenvironment.

Collectively, these studies demonstrate that ELISPOT provides a functional readout of immune suppression reversal, antigen validation, and therapy-induced T cell activation across diverse checkpoint-related interventions. Beyond confirming immune enhancement, the assay reveals cross-reactive immunity, dynamic modulation of antigen-specific populations, and the predictive value of baseline immune competence. Its ability to detect both restoration of effector function and persistence of cytotoxic capacity within the tumor microenvironment underscores its relevance for evaluating and optimizing immune checkpoint-based therapies.

Taken together, these studies demonstrate that ELISPOT is consistently used to quantify functional T cell responses following immune checkpoint modulation. However, variability in response magnitude, differences in treatment regimens, and inconsistent reporting of assay parameters limit cross-study comparability. Notably, although immune activation is frequently observed, its association with clinical outcomes remains inconsistent, underscoring the need for standardized methodologies and longitudinal validation.

#### 3.3.5. Biomarkers

Biomarker identification is a central objective in oncology, enabling improved disease detection, patient stratification, therapeutic monitoring, and prognostic assessment. Within this context, ELISPOT has emerged as a functional platform for quantifying antigen-specific T cell activity, thereby providing dynamic immunological information beyond static molecular markers.

**Figure 8:**
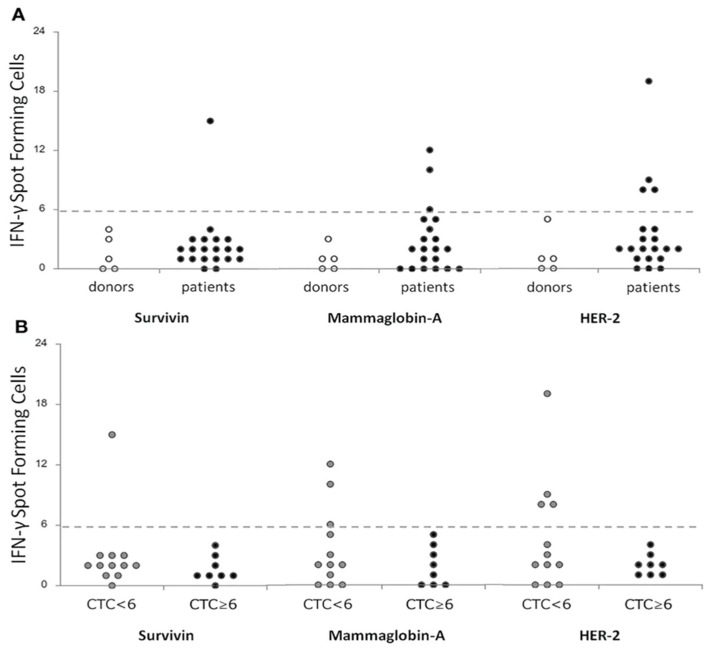
Correlation of T-cell specific responses against breast-tumor associated antigens (Survivin, Mammoglobin-A and HER2) between a cohort of donors (n = 5) and metastatic breast cancer patients (n = 20) before treatment (**A**) and between patients stratified according to the level of CTCs < 6 (n = 12) or ≥6 (n = 8) (**B**). T-cell responses were considered positive if at least 6 IFN-γ spot-forming cells were detectable. CTC, Circulating Tumor Cells; IFN-γ, Interferon-gamma. Reproduce from [109] under the terms and conditions of the Creative Commons Attribution (CC BY) license.

A translational study conducted at the Health Research Institute of Santiago de Compostela illustrated the relevance of functional immune biomarkers in breast cancer. Using ELISPOT, Muraro et al. [109] characterized T cell responses against the tumor-associated antigens Survivin, mammaglobin-A (MAM-A), and HER2 in patients with locally advanced disease. Importantly, the presence of antigen-specific responses correlated with increased T cell receptor (TCR) repertoire clonality and a reduced number of circulating tumor cells (CTCs), suggesting that functional immune activation may reflect improved tumor control and serve as a favorable prognostic indicator. Figure 8 shows a correlation of T-cell specific responses against breast-tumor associated antigens (Survivin, Mammoglobin-A and HER2) between a cohort of donors (n = 5) and metastatic breast cancer patients (n = 20) before treatment (Figure 8A) and between patients stratified according to the level of CTCs <6 (n = 12) or ≥6 (n = 8) (Figure 8B).

Beyond tumor-associated antigens, ELISPOT has also been applied to evaluate systemic immune competence during treatment. Liu et al. [110], at the Dana-Farber Cancer Institute, measured T cell responses against influenza A antigens and CEF—a peptide pool derived from cytomegalovirus (CMV), Epstein–Barr virus (EBV), and influenza virus—in patients undergoing neoadjuvant therapy. They observed progressive increases in antigen-specific T cell activity during treatment, indicating that ELISPOT can capture dynamic modulation of immune responsiveness over time.

The utility of the assay as a clinically relevant immune biomarker platform was further supported by [111,112], who independently investigated T cell responses to the spike protein in lung cancer patients. In these studies, ELISPOT enabled the functional assessment of antigen-specific immunity in contexts where both malignancy and therapeutic interventions may influence immune competence.

In colorectal cancer, ELISPOT has also contributed to the identification of negative or immunosuppressive biomarkers. Li et al. [113] examined T cell activity in patients with PD-L1-positive platelets and demonstrated reduced antigen-specific responses in this subgroup. These findings suggest that platelet-associated PD-L1 expression may contribute to systemic immune suppression and could represent a functional biomarker of diminished anti-tumor immunity.

Finally, ref. [114] from the University of Twente, evaluated immune responses in patients treated with a prostate-specific antigen (PSA)-based vaccine. ELISPOT analysis confirmed antigen-specific T cell activation, supporting the potential role of PSA as both a diagnostic marker and an immunological biomarker in therapeutic settings.

Collectively, these studies demonstrate that ELISPOT contributes to biomarker discovery across multiple dimensions of oncology. It enables identification of positive functional biomarkers—such as antigen-specific responses against Survivin, MAM-A, HER2, or PSA—while also revealing negative immune correlates, including reduced T cell activity associated with PD-L1-positive platelets. Moreover, its capacity to monitor longitudinal changes in immune responsiveness during treatment highlights its value in dynamic therapeutic assessment and clinical decision-making.

In response to recent advances in high-sensitivity biomarker detection, alternative automated and semi-automated platforms such as Quanterix SiMoA and Alamar NuLISA have emerged as state-of-the-art technologies for protein quantification [28,29,30]. When comparing these platforms to ELISPOT, the primary distinction lies like the data retrieved: functional competence versus absolute quantification. ELISPOT remains the “gold standard” for functional immunology because it captures the active secretion of cytokines from live, single cells, providing a direct link between an antigen stimulus and a cellular response [28,32]. In contrast, Quanterix SiMoA and Alamar NuLISA are “liquid biopsy” powerhouses designed to detect ultra-low concentrations of proteins already circulating in fluid (serum, plasma, or CSF). While Quanterix SiMoA and Alamar NuLISA offer unprecedented attomolar sensitivity and high-throughput automation, they provide a “snapshot” of systemic inflammation without identifying the specific cellular source or ensuring that the immune system is still capable of a coordinated response [28,29,30,31,32].

Furthermore, the platforms diverge significantly in their clinical scalability and multiplexing capabilities. Quanterix SiMoA and Alamar NuLISA utilize automated, bead-based or nucleic acid-linked workflows that minimize human error and are easily integrated into routine pathology labs using stable, cell-free samples [30,31]. ELISPOT, however, faces a higher translational barrier due to its requirement for viable peripheral blood mononuclear cells (PBMCs), making it sensitive to pre-analytical variables such as freezing and thawing protocols [32]. While Alamar NuLISA, in particular, excels at high-plex profiling—allowing researchers to monitor hundreds of biomarkers simultaneously—traditional ELISPOT is often limited to a few readouts. The future of the field likely lies in the “middle ground” currently being paved by nanotechnology-enhanced functional assays, which aim to combine the deep biological insights of cellular secretion with the precision, multiplexing, and automation of the digital proteomic era [30,34].

### 3.4. ELISPOT as a Functional Biomarker Versus a Laboratory Readout: A Critical Synthesis

To assess the role of ELISPOT as a functional biomarker in a rigorous translational sense, it is necessary to operationalize the concept. According to the FDA-NIH Biomarker Working Group BEST (Biomarkers, EndpointS, and other Tools) framework, biomarkers can be classified into four main categories based on their clinical function. Diagnostic biomarkers distinguish individuals with a condition from those without; prognostic biomarkers predict the natural course of disease independently of treatment; predictive biomarkers identify individuals likely to respond to a specific therapeutic intervention; and monitoring biomarkers assess the status of a disease or its response to treatment over time. Additional dimensions of biomarker validation include analytical validity, encompassing reproducibility, interlaboratory variability, and cut-off values, as well as clinical utility, defined as the capacity to inform clinical decisions and improve patient outcomes.

Applying this framework to the 78 studies reviewed, ELISPOT demonstrates a clearly defined role as a monitoring biomarker in the majority of cases, capturing dynamic changes in antigen-specific T cell activity during or following therapeutic intervention. Its role as a predictive biomarker, identifying patients likely to benefit from a specific therapy, is supported by a smaller but clinically significant subset of studies. Evidence for its use as a prognostic or diagnostic biomarker remains limited and requires further prospective validation. Notably, analytical validity, including standardized cut-off values and interlaboratory reproducibility, has not been formally established in most of the reviewed studies, representing a critical gap between the current research use of ELISPOT and its potential clinical implementation. Although ELISPOT is widely employed as a functional readout of antigen-specific immune responses and is frequently proposed as a biomarker in cancer immunotherapy, our analysis reveals that most studies lack standardized assay criteria (Table S2), which may limit its reproducibility and clinical translation.

A critical appraisal of the 78 studies included in this review reveals an important distinction that has not been explicitly addressed in previous literature: in the vast majority of cases, ELISPOT functions as a laboratory readout; that is, it confirms that an immunological event has occurred (e.g., antigen-specific IFN-γ secretion following vaccination or treatment), but does not establish a direct correlation with clinical outcomes such as overall survival, tumor burden reduction, or disease-free survival. Only a minority of the reviewed studies, approximately six out of 78, employed ELISPOT in a manner consistent with its role as a true functional biomarker, defined here as a measurement that is prospectively or retrospectively associated with a clinically meaningful endpoint.

Among these, Rocconi et al. [49] demonstrated that IFN-γ secretion in patients with recurrent ovarian cancer treated with the Vigil vaccine was associated with improved long-term survival, representing one of the clearest examples of ELISPOT as a predictive biomarker in this review. Similarly, ref. [62] reported that CTL responses against LMP2 in nasopharyngeal carcinoma correlated with improved five-year survival outcomes. In castration-resistant prostate cancer, ref. [105] showed that pre-treatment lymphocyte functionality measured by ELISPOT predicted lower tumor burden following six cycles of radium-223 therapy, suggesting a prognostic role for baseline immune competence. In breast cancer, Muraro et al. [109] correlated antigen-specific T cell responses against Survivin, MAM-A, and HER2 with increased TCR repertoire clonality and reduced circulating tumor cell counts, indicating that functional immune activation may reflect improved tumor control. In colorectal cancer, Li et al. [113] identified platelet-associated PD-L1 expression as a negative functional biomarker, as it was associated with diminished antigen-specific T cell responses. Finally, ref. [104] captured dynamic longitudinal modulation of TERT-specific CD4^+^ Th1 cells during chemoimmunotherapy in non-small-cell lung cancer, illustrating ELISPOT’s potential for monitoring immune fluctuations over time.

In contrast, the remaining studies, spanning cancer vaccines, oncolytic viruses, cellular therapies, and immune checkpoint approaches, predominantly employed ELISPOT to confirm immune activation without formally linking the magnitude or quality of the detected response to clinical benefit. While this use is scientifically valid and essential for mechanistic understanding and platform optimization, it does not meet the criteria for classifying ELISPOT as a validated functional biomarker in those contexts.

This distinction has important implications for the field. The transition of ELISPOT from a research tool to a clinically actionable biomarker requires prospective studies that correlate ELISPOT-measured immune responses with patient outcomes, standardization of assay protocols across institutions, and integration with complementary platforms such as high-dimensional flow cytometry or single-cell transcriptomics. Until these conditions are met, ELISPOT should be regarded as a powerful and sensitive laboratory readout that informs therapeutic development, rather than a validated prognostic or predictive biomarker in most oncological contexts.

Across studies, variability in ELISPOT standardization, incomplete reporting of positivity thresholds, and differences in study design introduce potential sources of bias that limit direct comparability, as detailed in Table S2.

### 3.5. ELISPOT Sample Types

The most commonly used sample is peripheral blood mononuclear cells (PBMCs), isolated from fresh or cryopreserved peripheral blood by density-gradient centrifugation (e.g., Ficoll-Paque) [28,29]. In addition, ELISPOT can be performed with whole blood (using specialized “whole-blood ELISPOT” protocols), spleen-, lymph-node-, or mucosal-tissue-derived cells (e.g., lamina propria lymphocytes) in animal or human tissue studies, and purified cell subsets (e.g., CD4^+^, CD8^+^ T cells, NK cells, or B-cell lines) when the experimental question targets a specific lineage [28,29,30,31]. In contrast, cell-free fluids such as plasma, serum, CSF, or conditioned media are not adequate ELISPOT samples, as they lack intact secreting cells and should instead be analyzed by ELISA or multiplex immunoassays. When selecting a sample, three main factors should be considered: cell type, viability, and functional capacity [31,32]. For most human T- or B-cell functional assays, PBMCs are the preferred default because they include antigen-presenting cells (APCs) that can process and present soluble antigens to T cells, enabling proper stimulation [32]. Fresh PBMCs are optimal, but cryopreserved PBMCs can also be used if they are thawed carefully, rested for about 1 h, and washed to remove cell debris before plating [33,34]. Cell source should be tailored to the immunological question: peripheral blood PBMCs are appropriate for systemic T-cell responses (e.g., vaccine studies); tissue-derived lymphocytes (e.g., tumor-infiltrating lymphocytes) capture local or organ-resident responses; and PBMCs or bone-marrow-derived B-cell-enriched preparations are suitable for antibody-secreting-cell assays [34]. A typical ELISPOT setup requires a minimum of 1–3 × 10^5^ cells per well, so the original blood or tissue volume must be sufficient to yield enough cells for triplicate wells and controls [32]. Each sample type carries practical and biological limitations that can affect sensitivity, reproducibility, and interpretation. PBMCs offer standardized isolation and a good representation of circulating T and B cells, but isolation can introduce variability (e.g., loss of certain subsets), and high proportions of dead or apoptotic cells reduce reliability and increase background noise [30,31]. Whole blood avoids PBMC isolation and minimizes manipulation-induced artifacts, making it useful when blood volume is limited; however, it often shows stronger background from granulocytes and other non-target cells, lower sensitivity than purified PBMCs, and requires optimized red-blood-cell lysis and specialized protocols [30,31]. Tissue-derived cells (e.g., spleen, lymph nodes, mucosa) capture local immune responses that may not be visible peripherally. Still, isolation is more complex, yields can be low, viability may be poorer, and the APC:T cell ratio may be skewed, potentially impairing antigen-driven responses. Purified cell subsets allow precise study of a given lineage, but removal of APCs can impair antigen-induced T-cell responses unless exogenous APCs or antigen-presenting support is added; in addition, magnetic or FACS-based purification can activate cells and alter basal secretion [35].

Some sample types are not adequate for ELISPOT. Cell-free fluids alone (plasma, serum, CSF, conditioned media) cannot serve as the primary ELISPOT sample because they lack secreting cells and will yield no spots even if the cytokine concentration is high [30,34]. Fixed or heavily dead cell preparations (e.g., formalin-fixed PBMCs or thawed samples with >30–40% dead cells) are likewise inadequate, as dead cells do not secrete cytokines and can increase background and false positives. Samples with very low cell counts (e.g., <10^5^ total PBMCs across all wells) may not support reliable spot counts, particularly when replicate wells and controls are required [35,36].

## 4. Conclusions

The 78 studies included in this systematic review collectively demonstrate the broad utility of ELISPOT as a highly sensitive functional assay for assessing antigen-specific cellular immune responses across major research areas in cancer immunotherapy, including cancer vaccines, oncolytic viruses, cellular therapies, immune checkpoint inhibitors, and biomarker development. Its single-cell resolution, flexibility in antigen selection, and capacity to capture dynamic immune responses make it a valuable tool for mechanistic and translational research.

However, a critical synthesis of the available evidence indicates that ELISPOT currently operates predominantly as a laboratory readout rather than a clinically validated functional biomarker in most oncological contexts. Only a limited subset of studies established a direct, statistically significant association between ELISPOT responses and clinical outcomes, such as overall survival, tumor burden, or circulating tumor cell dynamics. Importantly, our analysis further reveals that the majority of studies lack standardized assay criteria, including clearly defined positivity thresholds and reproducibility parameters (Table S2), which represents a major limitation for cross-study comparability and hinders clinical translation.

These findings highlight that, while ELISPOT is a powerful platform for detecting antigen-specific immune activation, its implementation as a clinically actionable biomarker requires rigorous standardization, prospective validation, and integration with complementary high-dimensional approaches. Addressing these challenges will be essential to bridge the gap between experimental immunomonitoring and clinical decision-making, ultimately supporting its potential integration into precision oncology frameworks.

## 5. Future Directions

Despite its versatility and significant applications in cancer research and other diseases, ELISPOT has several limitations, including relatively long execution times, the need for cell culture, and the requirement for specialized personnel. In addition, because each assay measures only a limited number of variables, it is restricted to quantifying cells secreting specific cytokines. However, combining ELISPOT with multiparametric techniques, such as single-cell transcriptomics or high-dimensional flow cytometry, may provide a more comprehensive characterization of immune responses. Furthermore, standardized procedures across laboratories have not yet been fully established, as protocol variability hampers the comparability of results and the validation of reliable biomarkers.

Future developments should focus on the design of simplified and accessible platforms capable of reliably measuring cellular immunity without the need for large equipment, specialized facilities, or highly trained personnel. In this context, the development of portable and user-friendly ELISPOT-based systems represents a promising direction. In parallel, advances in multiplexing approaches may enable simultaneous assessment of antigen specificity and cellular functional profiles, thereby enhancing the assay’s analytical depth. Addressing these challenges could facilitate the broader application of ELISPOT in clinical settings, while maintaining its value as a research tool.

## Data Availability

No new data were created or analyzed in this study. Data sharing is not applicable to this article.

## Supplementary Materials

The following supporting information can be downloaded at: https://www.mdpi.com/article/10.3390/ijms27094056/s1.

## Abbreviations

The following abbreviations are used in this manuscript:

Ad	Adenovirus
ALDH	Aldehyde Dehydrogenase
anti-CTLA-4	Anti-Cytotoxic T-Lymphocyte Antigen 4 antibody
APC	Antigen-Presenting Cell
ARG1	Arginase 1
BCG	Bacillus Calmette-Guérin
CAdVEC	Conditional Adenovirus Vector
CALR	Calreticulin
CALRLong36	CALR-mutant long peptide of 36 amino acids
CEF	Cytomegalovirus, Epstein–Barr virus, and Flu
ChAdOx1	Chimpanzee Adenovirus Oxford 1
circRNA	Circular RNA
CMV	Cytomegalovirus
CRT	Chemoradiotherapy
CSC	Cancer stem cell
CTC	Circulating tumor cells
CTL	Cytotoxic T Lymphocyte
DC	Dendritic cell
dLN	Draining lymph node
EBV	Epstein–Barr virus
EGFR	Epidermal Growth Factor Receptor
ELISA	Enzyme-Linked Immunosorbent Assay
ELISPOT	Enzyme-Linked ImmunoSpot Assay
ESR1	Estrogen Receptor 1
GEM	Gemcitabine
GSC	Glioma stem cell
Hepa	Hepatocellular carcinoma cell line
HER2	Human Epidermal Growth Factor Receptor 2
HNSCC	Head and Neck Squamous Cell Carcinoma
HPV	Human Papillomavirus
IFN-γ	Interferon gamma
iPSDCs-CTOS	Induced Pluripotent Stem Cell-derived Dendritic Cells–Cancer Tissue-Originated Spheroids
iPSL/DCs	Induced pluripotent stem cell-derived dendritic cells
IRE	Irreversible electroporation
iRFA	Incomplete Radiofrequency Ablation
ivtRNA	In vitro RNA
KIR	Killer-cell Immunoglobulin-like Receptors
LCL	Lymphoblastoid Cell Line
LDHC	Lactate Dehydrogenase C
LMP2	Latent Membrane Protein 2
MAM-A	Mammaglobin-A
mDC	Mature Dendritic Cell
MDSC	Myeloid-Derived Suppressor Cells
MiOncoCirc	Metastatic breast cancer RNA Onco-Circular database
MOC2	Murine Oral Carcinoma 2
MRB	Measles Virus Edmonston strain Receptor-Binding
MT	Mutant Type
MVA	Modified Vaccinia Ankara
NAM	Nicotinamide
NCL	Nucleolin
NK	Natural killer
NSCLC	Non-Small-Cell Lung Cancer
OV	Oncolytic Virus
OVA	Ovalbumin
PAP	Prostatic Acid Phosphatase
PBMC	Peripheral Blood Mononuclear Cells
PD-1	Programmed cell death protein 1
PD-L1	Programmed death-ligand 1
PDT	Photodynamic therapy
PGE2	Prostaglandin E2
PIK3CA	Phosphatidylinositol-4,5-bisphosphate 3-kinase catalytic subunit alpha
PPM1F	Protein Phosphatase, Mg2^+^/Mn2^+^ Dependent 1F
PSA	Prostate-Specific Antigen
PSAP	Prostatic specific acid phosphatase
qPCR	Quantitative Polymerase Chain Reaction or Real-Time Polymerase Chain Reaction
rBCG-S.FimH	Recombinant Bacillus Calmette-Guérin expressing FimH
rhIL-7	Recombinant human Interleukin-7
SV.IL12	Sindbis Virus expressing Interleukin 12
TAA	Tumor-Associated Antigen
TCR	T cell receptor
TCR-T	T cell receptor-engineered T cells
TERT	Telomerase Reverse Transcriptase
TIL	Tumor-Infiltrating Lymphocyte
Treg	Regulatory T Cell
VEGF	Vascular Endothelial Growth Factor
WT	Wild Type
αOX40	Anti-OX40 antibody (alpha-OX40)
