# Supplementary material for: ELISPOT as a Functional for Biomarker Study in Cancer Immunotherapy: Applications and Future Directions
Source: Int J Mol Sci. 2026 Apr 30;27(9):4056. doi: 10.3390/ijms27094056 (PMC13164536; doi:10.3390/ijms27094056)
Supplement: Supplementary file 1 [file ijms-27-04056-s001.zip › ijms-4215596-supplementary.pdf]

Table S1. PRISMA 2020 Checklist.

Section	Item	Description	Location in manuscript
Title	1	Identify the report as a systematic review	Title
Abstract	2	Structured summary of objectives, methods, and findings	Abstract
Introduction	3	Rationale for the review	Section 1
Introduction	4	Objectives of the review	Section 1
Methods	5	Eligibility criteria (inclusion/exclusion)	Section 2.2
Methods	6	Information sources (databases)	Section 2.1
Methods	7	Search strategy	Section 2.1
Methods	8	Selection process (screening)	Section 2.2
Methods	9	Data collection process	Section 2.5
Methods	10	Data items extracted	Table S1
Methods	11	Risk of bias assessment (not performed; justification provided)	Section 2.4
Methods	13	Synthesis methods (qualitative synthesis)	Section 2.3
Results	16	Study selection process (flow diagram)	Figure 2
Results	17	Study characteristics	Table S1
Discussion	23	Limitations of the review	Section 2.4 and Discussion
Discussion	24	Interpretation of results	Sections 3 and 4
Other	27	Availability of supporting data	Supplementary Material

Table S2. Summary of studies included in the systematic review evaluating ELISPOT as a functional biomarker in cancer immunotherapy.

	First Author/ Year	Cancer Type	Study Design	Research Line	ELISPOT Readout	Cell Population	Antigen/Stimulation	Positivity Threshold	Clinical Outcome Association	Biomarker Category	ELISPOT Standardization Level
[42]	Wang et al., 2020	Bladder cancer	Clinical	Cancer vaccines	IFN-γ	PBMC (HLA-A2+ patients)	Wild-type and mutant peptides from exomic sequencing	Not reported	No	Monitoring	Low
[43]	Mistretta et al., 2023	Breast cancer	Clinical	Cancer vaccines	IFN-γ	PBMC / CD8+ T cells (HLA-C*07:02 matched healthy donor)	15 neoantigen peptides from NSFP1-LRRC37A2 fusion transcript	≥20 SFC/10^6 cells above background	No	Monitoring	High
[44]*	Zhang et al., 2024	Triple-negative breast cancer	Clinical (Phase 1 trial)	Cancer vaccines	IFN-γ	PBMC	Personalized neoantigen overlapping peptides (median 11 neoantigens/patient)	Statistical significance $p<0.05$ (Student's t-test)	Yes (recurrence-free survival 87.5% at 36 months)	Predictive	Moderate
[45]	Morisaki et al., 2024	Breast cancer	Clinical (retrospective)	Cancer vaccines	IFN-γ	PBMC / Lymphocytes	HLA class II-restricted neoantigen long peptides pulsed onto autologous dendritic cells	Not reported explicitly	Yes (no recurrence in all 5 patients)	Monitoring	Low

	First Author/ Year	Cancer Type	Study Design	Research Line	ELISPOT Readout	Cell Population	Antigen/Stimulation	Positivity Threshold	Clinical Outcome Association	Biomarker Category	ELISPOT Standardization Level
[46]	Lian et al., 2024	Lung cancer	Preclinical (murine)	Cancer vaccines	IFN-γ	Splenocytes	Translatome-derived neoantigen peptides vs. routine neoantigen peptides	Statistical significance $p<0.05$ vs. unstimulated	No	Monitoring	Moderate
[47]	Corulli et al., 2021	Colorectal cancer	Clinical + Preclinical (murine)	Cancer vaccines	IFN-γ	PBMC (CRC patients and healthy donors)	CDC25B, COX2, FASCIN1, RCAS1 peptides (10 μg/ml) + recombinant proteins (1 μg/ml)	Statistical significance vs. negative control (HIVp52), $p<0.05$	No	Monitoring	Moderate
[48]	Yu et al., 2022	Colorectal cancer	Clinical + Preclinical (mixed)	Cancer vaccines	IFN-γ	PBMC / Peripheral blood lymphocytes + Splenocytes (murine)	Mutant neoantigen peptides (HAVCR2-F39V, SEC11A-R11L, ULK1-S248L) pulsed on autologous DCs	Not reported ($p<0.01$ for comparative analysis)	No	Monitoring	Low
[49]	Schwarz et al., 2022	Colorectal cancer (high TMB, MSI)	Clinical	Cancer vaccines	IFN-γ	PBMC (peripheral T cells) + TIL	97 cryptic peptides + 3 classically mutated	Not reported	No	Monitoring	Low

	First Author/ Year	Cancer Type	Study Design	Research Line	ELISPOT Readout	Cell Population	Antigen/Stimulation	Positivity Threshold	Clinical Outcome Association	Biomarker Category	ELISPOT Standardization Level
						(tumor-infiltrating T cells)	neoantigens (IQGAP1, CTNNB1, TRIT1) presented via B-LCL				
[50]	Zhang et al., 2024	Colorectal cancer	Clinical	Cancer vaccines	IFN-γ	PBMC / CD8+ T cells (patient-derived, DC-mediated presentation)	MHC class I-restricted 9aa neoantigen peptides (WES-predicted) + CpG adjuvant	Not reported (spot counts recorded as T-value per patient)	No	Monitoring	Low
[51] *	Rocconi et al., 2021	Ovarian cancer (recurrent)	Clinical (Phase I/II trials)	Cancer vaccines	IFN-γ	PBMC	Autologous tumor cell antigens (Vigil vaccine)	Not reported explicitly	Yes (OS: median not reached in ELISPOT+ vs. 9.5 months in ELISPOT–, $p=0.0079$)	Predictive	Low
[52]	Liu et al., 2024	Ovarian cancer	Preclinical (rat model)	Cancer vaccines	IFN-γ / IL-4 (dual-color)	Splenocytes	TSA-Nutu-R vaccine (TSA-modified irradiated Nutu-19 tumor cells)	Statistical significance $p<0.01$ vs. controls	No	Monitoring	Moderate

	First Author/ Year	Cancer Type	Study Design	Research Line	ELISPOT Readout	Cell Population	Antigen/Stimulation	Positivity Threshold	Clinical Outcome Association	Biomarker Category	ELISPOT Standardization Level
[53]	Pachynski et al., 2021	Prostate cancer (mCRPC)	Clinical (Phase II trial)	Cancer vaccines / Immune checkpoint	IFN-γ	PBMC	PA2024 (50 μg/ml) + PAP (25 μg/ml)	Not reported explicitly (cSPW background-subtracted)	No (no significant differences between rhIL-7 and observation groups by ELISPOT)	Monitoring	Low
[54]	Geboers et al., 2025	Prostate cancer (localised)	Clinical (prospective)	Cancer vaccines / Immune checkpoint	IFN-γ	PBMC	Prostatic acid phosphatase (PSAP) + NY-ESO-1 cancer testis antigen	Not reported	No	Monitoring	Low
[55]	Selvanesan et al., 2020	Pancreatic cancer	Preclinical (murine, orthotopic Panc-02 and KPC models)	Cancer vaccines	IFN-γ	Splenocytes	Survivin TAA (pcDNA-3.1-survivin transfection)	Not reported	No	Monitoring	Low
[56] ‡	Nakazawa et al., 2022	Colorectal cancer / Breast cancer (HLA-A33 matched)	Preclinical (in vitro, human cell lines)	Cellular therapy	IFN-γ	CTL (CD8+ T cells derived from PBMC, HLA-A33 donor)	iPSC lysate-pulsed dendritic cells vs. autologous tumor cell lines (SW48, T47D)	Statistical significance $p<0.05$ (Mann-Whitney U test)	No	Monitoring	Moderate
[57]	da Silva et al., 2020	Cervical cancer (locally	Clinical (Phase I trial,	Immune checkpoint	IFN-γ	PBMC	HPV16 and HPV18 E6/E7 long	>50 IFNγ-secreting cells per	No (cytokines associated with PFS, but ELISPOT not	Monitoring	High

	First Author/ Year	Cancer Type	Study Design	Research Line	ELISPOT Readout	Cell Population	Antigen/Stimulation	Positivity Threshold	Clinical Outcome Association	Biomarker Category	ELISPOT Standardization Level
		advanced, HPV+)	GOG-9929)				overlapping peptide pools	million PBMC	directly correlated)		
[58]	Akazawa et al., 2020	Non-small cell lung cancer (EGFR T790M/C797S)	Preclinical (in vitro, healthy HLA-A*02:01 donors)	Cancer vaccines	IFN-γ	PBMC / CD8+ CTL (HLA-A*02:01 healthy donors)	EGFR T790M/C797S-derived peptide (MQLMPFGSLL, 10 μg/ml) pulsed on T2 cells	Not reported explicitly	No	Monitoring	Low
[59] §	Thomas et al., 2020	Breast cancer (LDHC-expressing tumors)	Preclinical (in vitro, PBMC from healthy donors)	Cancer vaccines	IFN-γ	PBMC / CD8+ T cells (HLA-A*0201 healthy donors) + breast cancer cell co-cultures	LDHC-derived peptide pools (PP2, PP8) + DCs pulsed with individual peptides	SFU/10^6 cells ≥ 100 with PP/control ratio ≥ 3	No	Monitoring	High
[60]	Handlos Grauslund et al., 2021	Myeloproliferative neoplasms (CALR-mutant)	Clinical (Phase I trial, NCT03566446)	Cancer vaccines	IFN-γ	PBMC	CALRLong36 peptide (200 μg, 36-aa mutant CALR C-terminus)	Not reported explicitly (background-subtracted spot counts)	No (no clinical responses despite T cell responses)	Monitoring	Low
[61]	Gao et al., 2020	TNBC	Preclinical (murine; 4T1, EMT6, E0771)	Cancer vaccines / Immune checkpoint	IFN-γ	Tumor-infiltrating CD8+ T cells	Tumor cell antigens (CBDCA + anti-PD-1	Not reported	No	Monitoring	Low

	First Author/ Year	Cancer Type	Study Design	Research Line	ELISPOT Readout	Cell Population	Antigen/Stimulation	Positivity Threshold	Clinical Outcome Association	Biomarker Category	ELISPOT Standardization Level
							treatment context)				
[62]	McCann et al., 2022	NSCLC (TMB-low)	Clinical (case study, single patient)	Cancer vaccines	IFN-γ	PBMC (autologous, 13-day in vitro stimulation + IL-2)	Mutated long peptides (KEAP1, KIAA0408, POC1B, NIF3L1, MAFF)	Not reported	No	Monitoring	Low
[63]	Poole et al., 2022	Pancreatic / Colorectal cancer (KRASG12D)	Preclinical (in vitro, PBMC from healthy donors)	Cellular therapy	IFN-γ	PBMC (healthy donors, HLA-A*11:01+)	KRASG12D-derived decamer peptide (VVVGADGVGK, 10 μM) via IMC-KRASG12D bispecific TCR-CD3 molecule	Not reported (EC50 values used for dose-response)	No	Monitoring	Low
[64] ‡	Zeng et al., 2020	Nasopharyngeal carcinoma (EBV+)	Clinical (Phase I/II trial, n=29)	Cancer vaccines	IFN-γ	PBMC (patients, 2×10^5 cells/well)	EBV-LMP2 peptide sub-pools (5 μg/mL), via LMP2-DC vaccine	Not reported	No (ELISPOT used to confirm immunogenicity; 5-year survival data available but not correlated with ELISPOT)	Monitoring	Low
[65]	Chen et al., 2021	Pancreatic cancer	Clinical (prospect	Cancer vaccines	IFN-γ	PBMC (patients,	Personalized neoantigen	Not reported (spot counts	No (tumor regression	Monitoring	Low

First Author/ Year		Cancer Type	Study Design	Research Line	ELISPOT Readout	Cell Population	Antigen/Stimulation	Positivity Threshold	Clinical Outcome Association	Biomarker Category	ELISPOT Standardization Level
		(advanced, refractory)	ive, personalized neoantigen vaccine)			2×10⁵ cells/well, 16-24h ex vivo)	peptides (iNeo-Vac-P01, predicted from WES)	at multiple timepoints post-vaccination)	documented in 1 patient; not formally correlated with ELISPOT)		
[66]	Jørgensen et al., 2021	Multiple cancers (syngeneic murine models)	Preclinical (in vivo, varios modelos singénicos murinos)	Cancer vaccines / Immune checkpoint	IFN-γ	Splenocytes (murine)	ARG1-derived peptide vaccines ± anti-PD-1	Not reported	No	Monitoring	Low
[67]	Cecil et al., 2021	Ovarian cancer (advanced)	Clinical (Phase I, n=25)	Cancer vaccines	IFN-γ / IL-10 (dual ELISPOT)	PBMC (patients, 4 replicates, 2×10⁵/well)	IGFBP-2 N-terminus class II binding peptide pool (10 μg/mL)	cSPW > 2 SD above pre-vaccination value	No (immunogenicity endpoint; no OS/PFS correlation reported)	Monitoring	High
[68] †	Morisaki et al., 2021	Ovarian cancer (chemorefractory, recurrent)	Clinical (case report, n=1)	Cancer vaccines	IFN-γ	Lymphocytes from peripheral blood (5×10³ autologous immature DCs/well + neoantigen peptides	Personalized neoantigen peptides (4 predicted neoantigens, esp. PPM1F mutation, HLA-A2402-restricted)	Not reported	Yes (CA-125 decline, reduced tumor cells in ascites after 4 vaccination rounds) ☆	Monitoring	Low

	First Author/ Year	Cancer Type	Study Design	Research Line	ELISPOT Readout	Cell Population	Antigen/Stimulation	Positivity Threshold	Clinical Outcome Association	Biomarker Category	ELISPOT Standardization Level
						25 µg/mL, 4h)					
[69]	Feola et al., 2022	Colorectal cancer (CT26 murine model)	Preclinical (in vivo, murine)	Cancer vaccines (PeptiCRAd oncolytic vaccine platform)	IFN-γ	Splenocytes (murine)	MHC-I restricted peptides from CT26 tumor surface (immunopeptidomics + HEX pipeline)	Not reported	No	Monitoring	Low
[70]	Shou et al., 2022	Multiple cancers (28 patients, various types)	Clinical + Preclinical	Cancer vaccines / Immune checkpoint (RFA + neoantigen vaccination)	IFN-γ	PBMC (patients, 2×10^5/well, 16-24h ex vivo)	Personalized neoantigen peptides (iNeo-Vac-P01 from WES) ± RFA	Not reported	No (descriptive cohort; no formal ELISPOT-outcome correlation)	Monitoring	Low
[71]	Salvatori et al., 2022	Colorectal cancer (MC38 murine model)	Preclinical (in vivo, murine)	Cancer vaccines / Immune checkpoint	IFN-γ	Splenocytes (C57Bl/6 mice)	M8 DNA plasmid vaccine (8 MC38 neoantigens as 28-mers) ± anti-CTLA-4 / anti-PD-1	Not reported	No	Monitoring	Low
[72]	Maruoka et al., 2022	Colorectal cancer	Preclinical (in vitro, patient-derived cells, n=3)	Cellular therapy	IFN-γ	CTL from PBMC/patient-derived cells + iPSDC-	Tumor RNA-transfected iPSC-derived DCs (ivtRNA) + 12 candidate	Not reported	No	Monitoring	Low

	First Author/ Year	Cancer Type	Study Design	Research Line	ELISPOT Readout	Cell Population	Antigen/Stimulation	Positivity Threshold	Clinical Outcome Association	Biomarker Category	ELISPOT Standardization Level
						ivtRNA co-culture	neoantigen peptides				
[73]	Shafer et al., 2024	Breast cancer (metastatic, ESR1-mutant)	Preclinical (in vitro, healthy donors + MBC patients)	Cellular therapy (TCR T)	IFN-γ	PBMC (healthy donors + MBC patients, DC-expanded T cell lines)	MBCneo-pepmix: overlapping 15-mer peptides covering 13 driver mutations (AKT1, ESR1, PIK3CA, TP53)	Not reported	No	Monitoring	Low
[74]	Liu et al., 2022	Breast cancer (HER2+)	Preclinical (in vivo, murine)	Cancer vaccines / Nanoparticle platform	IFN-γ	Splenocytes (murine, fat pad lymph node cells)	Microparticulate HER2 cancer vaccine (p66 peptide, 10 μg/mL, 36h) embedded in surgical abdominal flap	Not reported	No	Monitoring	Low
[75]	Li et al., 2023	Multiple cancers (syngeneic murine, OVA/TAA)	Preclinical (in vivo, murine)	Cancer vaccines / Nanoparticle platform	IFN-γ	Splenocytes (murine)	TAA-conjugated protein nanoparticle (E2) + adjuvant; TAA peptides (10 μg/mL, overnight)	Not reported	No	Monitoring	Low

	First Author/ Year	Cancer Type	Study Design	Research Line	ELISPOT Readout	Cell Population	Antigen/Stimulation	Positivity Threshold	Clinical Outcome Association	Biomarker Category	ELISPOT Standardization Level
[76]	Heo et al., 2023	Melanoma / Colorectal cancer (B16F10-OVA, MC38 murine models)	Preclinical (in vivo, murine)	Cancer vaccines / TLR agonist adjuvant (L-Pampo™)	IFN-γ	Splenocytes (murine, C57BL/6)	OVA257-264 peptide (25 µg/mL) ± L-Pampo™ vs. Montanide ISA51, GM-CSF	$p<0.001$ vs. PBS/OVA alone	No	Monitoring	Moderate
[77]	Ren et al., 2024	Multiple cancers (pan-cancer; circRNA neoantigen discovery in patient samples and murine models)	Preclinical + Clinical (translational)	Cancer vaccines / Neoantigen (circular RNA-derived peptides)	IFN-γ	PBMC (patient-derived) / Splenocytes (murine)	CircRNA-derived neoantigen peptides (circRNA-encoded open reading frames)	Not reported	No	Monitoring	Low
[78]	Peters et al., 2024	Ovarian cancer (high-grade serous, HGSOC; ex vivo human ascites and murine ID8 model)	Preclinical (in vitro + in vivo murine)	Cancer vaccines / Oncolytic virus (MEM-288: CD40L + IFNβ)	IFN-γ	Splenocytes (murine) / Ascites-derived immune cells (human)	Oncolytic adenovirus MEM-288 (membrane-stable CD40L + IFNβ); tumor cell antigens	Not reported	No	Monitoring	Low

	First Author/ Year	Cancer Type	Study Design	Research Line	ELISPOT Readout	Cell Population	Antigen/Stimulation	Positivity Threshold	Clinical Outcome Association	Biomarker Category	ELISPOT Standardization Level
[79]	Ishizuka et al., 2024	Breast cancer (BALB-MC murine allogeneic transplantation model)	Preclinical (in vivo, murine)	Cancer vaccines / Photodynamic therapy / Nanoparticle platform (ICG-Lipo-PTX)	IFN-γ	Splenocytes (murine)	ICG-Lipo-PTX + PDT (abscopal immune effect); cytokine secretory capacity assessed by ELISPOT	Not reported	No	Monitoring	Low
[80]	Li et al., 2025	Multiple murine tumors (B16-OVA melanoma, MC38 and CT26 colorectal) + humanized CDX (MDA-MB-231 breast cancer)	Preclinical (in vivo murine + humanized xenograft)	Cancer vaccines / Nanoparticle platform / STING adjuvant (AECM@PC7A)	IFN-γ	Splenocytes (murine)	AECM@PC7A nanovaccine (IFN-γ-enriched tumor cell membranes + PC7A STING-activating polymer); OVA257-264 peptide for ELISPOT restimulation	Not reported	No	Monitoring	Low
[81]	Huang et al., 2025	Gastric cancer (YDN16 murine model;	Preclinical (in vivo, murine)	Cancer vaccines / Nanoparticle platform (HPPS nanovaccine + RADA32 peptide hydrogel + Mn^{2+})	IFN-γ	Splenocytes (murine, C57BL/6)	HPPS-OT1@RMn (OVA-based, model antigen) and HPPS-	Not reported	No	Monitoring	Low

	First Author/ Year	Cancer Type	Study Design	Research Line	ELISPOT Readout	Cell Population	Antigen/Stimulation	Positivity Threshold	Clinical Outcome Association	Biomarker Category	ELISPOT Standardization Level
		MG7-expressing)					MG7@RMn (MG7-related gastric cancer antigen peptide + CpG-ODN + Mn^{2+}; TLR9 + cGAS-STING activation)				
[82]	Del Campo et al., 2025	Multiple murine tumor models (MC38 colorectal, CT26 colorectal, TC-1 HPV-related, B16-OVA melanoma)	Preclinical (in vivo, murine)	Cancer vaccines / mRNA platform (LNP-mRNA + OligoDOMTM self-assembling sequence)	IFN-γ	Splenocytes (murine, 2.5×10^5 cells/well, 24h restimulation with tumor-specific peptides at 2 μg/mL)	Tumor neoepitope peptides (Reps1, Adpgk for MC38; Mitch1 CD8, Dhx35 CD4 for CT26; E7 for TC-1; OVA CD8/CD4 for B16-OVA) fused to OligoDOMTM in LNP-mRNA constructs	Not reported ($p<0.01$ vs. constructs without OligoDOMTM)	No	Monitoring	Moderate
[83]	Roy et al., 2021	Multiple murine tumor models (B16F10-OVA	Preclinical (in vivo, murine)	Cellular therapy / Oncolytic virus (Ad, MRB, VSV, VV prime-boost ± antigenic peptide)	IFN-γ	Splenocytes (murine; ex vivo restimulation with tumor	Oncolytic virus (Ad, Maraba MRB, VSV, VV) co-administered with tumor	Not reported ($p<0.001$ vs. unstimulated "No restim" control)	No	Monitoring	Moderate

First Author/ Year		Cancer Type	Study Design	Research Line	ELISPOT Readout	Cell Population	Antigen/Stimulation	Positivity Threshold	Clinical Outcome Association	Biomarker Category	ELISPOT Standardization Level
		melanoma, others)				antigen or viral peptides)	antigenic peptides (DCT, OVA, E6/E7) or virus encoding tumor antigen (Ad-DCT, MRB-Ova, etc.)				
[84]	Shi et al., 2021	Breast cancer (4T1) and lung cancer (A549) murine models	Preclinical (in vivo, murine)	Cellular therapy / Oncolytic virus (ΔTK-Armed-VACV encoding anti-PD-1 + anti-4-1BB antibodies)	IFN-γ	Splenocytes (murine; ex vivo restimulation with tumor cells)	ΔTK-Armed-VACV (thymidine kinase-deleted oncolytic vaccinia virus expressing anti-human-PD-1 and anti-human-4-1BB double antibody genes; Western Reserve strain)	Not reported	No	Monitoring	Low
[85]	Ding et al., 2022	Colorectal cancer (CT26 murine model; pre-immunized BALB/c ±	Preclinical (in vivo, murine)	Cellular therapy / Oncolytic virus (novel immune-stimulating oncolytic HSV + pre-existing anti-	IFN-γ	Splenocytes (murine, 1×10^5 cells/well; overnight stimulation with	CT26 tumor cells (used to detect CT26-specific T cell responses) ± prior HSV-1	Not reported (spots per well)	No	Monitoring	Low

	First Author/ Year	Cancer Type	Study Design	Research Line	ELISPOT Readout	Cell Population	Antigen/Stimulation	Positivity Threshold	Clinical Outcome Association	Biomarker Category	ELISPOT Standardization Level
		prior HSV-1 immunity)		HSV-1 immunity)		CT26 tumor cells 5×10³/well)	immune background				
[86]	Morita et al., 2024	Multiple solid tumors (advanced cancer patients)	Clinical + Preclinical (humanized mice, patient samples)	Oncolytic virotherapy (CAdVEC/CAdTrio/CAdTetra – oncolytic + helper adenovirus with IL-12, PD-L1 blocker, BiTE anti-CD44v6)	IFN-γ	PBMCs from treated patients; splenocytes from humanized mice	Adenovirus-specific peptides; tumor cell lysate (Hepa1-6/tumor lines)	Not reported	Yes (complete response observed in patients)	Monitoring	Low
[87]	Martinez-Perez et al., 2024	HPV-16-induced cancer (TC-1 murine model – cervical cancer)	Preclinical (in vivo, C57BL/6 mice)	Cancer vaccines (oncolytic adenovirus encoding SP-SA-E7-4-1BBL)	IFN-γ	Splenocytes (C57BL/6 mice)	HPV-16 E7 antigen (ex vivo antigenic stimulation)	Not reported	No	Efficacy / Monitoring	Low
[88]	Sun et al., 2025	Hepatocellular carcinoma (HCC)	Preclinical (in vitro + murine orthotopic HCC model – Hepa1-6)	Oncolytic peptide + checkpoint inhibitor (LTX-315 + anti-CTLA-4) after radiofrequency ablation	IFN-γ (cytotoxic T cell activity)	CD8+ T cells extracted from Hepa1-6 tumors (murine)	Hepa1-6 tumor cells (co-culture with extracted CD8+ T cells + anti-CTLA-4)	Not reported	No	Efficacy	Low
[89]	Opp et al., 2025	Pancreatic cancer	Preclinical	Oncolytic virotherapy	IFN-γ	Splenocytes (murine;	No additional stimulus	Not reported	No	Efficacy /	Low

	First Author/ Year	Cancer Type	Study Design	Research Line	ELISPOT Readout	Cell Population	Antigen/Stimulation	Positivity Threshold	Clinical Outcome Association	Biomarker Category	ELISPOT Standardization Level
			(orthotopic murine models – three model systems)	(Sindbis virus-IL-12 + anti-OX40)		8×10^4 T cells/well)	(spontaneous IFN-γ; PMA/ionomycin as positive control)			Monitoring	
[90]	van Pul et al., 2020	Breast cancer (ex vivo sentinel lymph node samples)	Translational / ex vivo (breast cancer patient SLN single-cell suspensions, N=29)	Immunomodulation (CpG-B + JAK2/STAT3 inhibitor AG490)	IFN-γ	SLN-derived single-cell suspensions from breast cancer patients (N=29)	Mammaglobin-A overlapping 15-mer peptide pool (BrC-associated antigen)	Not reported	No	Monitoring	Low
[91]	Thongchot et al., 2022	Triple-negative breast cancer (TNBC)	Preclinical (in vitro; TNBC cell lines MDA-MB-231, HCC70)	Adoptive cell therapy (SmartDCs-NCL + anti-PD-L1 peptide → NCL-specific T cells)	IFN-γ	NCL-specific T cells activated by SmartDCs-NCL+RPS3 (from donor PBMCs); 110 T lymphocytes/well	NCL (nucleolin) peptides	Not reported (spots counted with BIOREADER 5000 Pro F)	Yes (NCL high / PD-L1 high associated with worst OS in 144 TNBC patients)	Predictive / Efficacy	Low

	First Author/ Year	Cancer Type	Study Design	Research Line	ELISPOT Readout	Cell Population	Antigen/Stimulation	Positivity Threshold	Clinical Outcome Association	Biomarker Category	ELISPOT Standardization Level
[92]	Gao et al., 2021	Breast cancer (TNBC; 4T1 murine model)	Preclinical (in vivo, BALB/c mice with large established 4T1 tumors)	Local immunochemotherapy (paraneoplastic CpG + α-OX40 + anthracycline)	IFN-γ	Splenocytes (BALB/c murine)	4T1 tumor cells + neoantigen epitopes (ex vivo stimulation by IFN-γ ELISpot and intracellular cytokine assay)	Not reported	No	Efficacy / Monitoring	Moderate
[93]	Liao et al., 2022	Melanoma (D5 murine model; ALDHhigh cancer stem cells)	Preclinical (in vivo, C57BL/6 mice with D5 melanoma; protection + therapeutic models)	Cancer vaccines (ALDH1A1/1A3 peptide-DC vaccine ± anti-PD-L1)	IFN-γ	Splenocytes (murine, from D5 melanoma-bearing mice)	ALDHhigh D5 CSCs vs. ALDHlow non-CSCs (ex vivo stimulation)	Not reported	No	Efficacy / Monitoring	Low
[94]	Miyaguchi et al., 2023	Glioblastoma (GBM; GSC lines from patients; HLA-A2+ donors)	Preclinical / Translational (in vitro; preparation for	Adoptive cell therapy (activated T cells [ATC] targeting GSC TAAs via DC loading: lysate, acid-	IFN-γ	T cells activated by antigen-loaded DCs (HLA-A2+	GSC lysate-pulsed DCs; GSC acid-eluate-pulsed DCs; GBM-specific synthetic TAA	Not reported	No (preclinical; Phase I planned)	Efficacy / Monitoring	Low

	First Author/ Year	Cancer Type	Study Design	Research Line	ELISPOT Readout	Cell Population	Antigen/Stimulation	Positivity Threshold	Clinical Outcome Association	Biomarker Category	ELISPOT Standardization Level
			planned Phase I clinical trial)	eluate, or synthetic peptide pool)		donor blood); T2 cells as targets	peptide pool (SOX2, HER2, AIM2, IL13Ra2, EphA2, etc.)				
[95]	Shirosaki et al., 2024	Gastric cancer (MKN-45 cell line; ALDHhigh cancer stem-like cells / CICs)	Preclinical (in vitro; ALDHhigh vs. ALDHlow gastric cancer cell clone co-culture)	Adoptive cell therapy (TCR-T cells targeting model neoantigen AP2S1; HLA-A24-restricted)	IFN-γ	AP2S1-specific TCR-T cells targeting ALDHhigh or ALDHlow MKN-45 clones	Model neoantigen AP2S1 overexpressed in ALDHhigh clone cells (HLA-A24)	Not reported	No	Efficacy	Low
[96]	Gustafson et al., 2025	Multiple metastatic epithelial cancers (colorectal, lung, breast, other solid tumors; N=263 harvests)	Clinical (TIL harvest and neoantigen screening, 2014–2023; N=291 operations; ongoing ACT clinical	Adoptive cell therapy (TIL neoantigen screening for ACT selection; lung lesions more likely to grow TIL)	IFN-γ (+ 4-1BB upregulation by flow cytometry)	TIL fragment cultures (up to 24/patient) co-cultured with autologous DCs electroporated with TMG or pre-incubated	Patient-specific tumor mutation tandem minigenes (TMG) + synthetic mutant peptide pools (PP) encoding all tumor mutations	Qualitatively positive ELISpot AND >20% 4-1BB upregulation; ≥2 reactive fragments required for ACT eligibility	Yes (reactive TIL selected for ACT; clinical responses observed in ongoing trial)	Predictive / Selection (for ACT)	High

	First Author/ Year	Cancer Type	Study Design	Research Line	ELISPOT Readout	Cell Population	Antigen/Stimulation	Positivity Threshold	Clinical Outcome Association	Biomarker Category	ELISPOT Standardization Level
			trial, NCI Surgery Branch)			with mutant peptide pools (PP)					
[97]	Greene et al., 2020	Head and neck squamous cell carcinoma (HNSCC; MOC2 murine model + HNSCC patients)	Preclinical + Translational (MOC2 murine model + HNSCC patient peripheral blood/tumor samples, N=12 donors)	NK cell immunotherapy + MDSC suppression (SX-682 CXCR1/2 inhibitor + adoptively transferred NK cells)	IFN-γ (NK cell activation; MDSC suppression assay)	Peripheral NK cells from HNSCC patients or healthy donors (N=12); murine NK cells from MOC2 tumor-bearing mice	NK cell stimulation (non-antigen-specific; ELISpot used to measure MDSC-mediated suppression of NK activation)	Not reported	No (preclinical rationale; precedes clinical development)	Efficacy / Monitoring	Low
[98]	Zhang et al., 2020	Multiple human cancers (pan-cancer; 9,700 TCGA bulk RNA-seq samples; validation	Computational + Preclinical validation (TRUST + iSMART TCR clustering	Biomarker discovery / TCR repertoire analysis (antigen-specific TCR cluster identification; novel cancer/testis	IFN-γ	Splenocytes from HLA-A*02:01 humanized mice vaccinated against HSFX1	HSFX1 peptide (novel cancer/testis antigen identified by iSMART/TRUST algorithm from TCR clustering analysis)	Not reported	No (computational/preclinical validation)	Biomarker discovery / Monitoring	Low

	First Author/ Year	Cancer Type	Study Design	Research Line	ELISPOT Readout	Cell Population	Antigen/Stimulation	Positivity Threshold	Clinical Outcome Association	Biomarker Category	ELISPOT Standardization Level
		in HLA-A*02:01 humanized mice)	algorithm; mouse vaccination for novel antigen validation)	antigen HSFX1 discovered)							
[99]	Sinha et al., 2021	Metastatic castration-resistant prostate cancer (mCRPC)	Clinical (Phase II randomized trial, NCT01804465; N=50 patients; immediate vs. delayed ipilimumab after sipuleucel-T)	Cancer vaccines + checkpoint inhibitor (sipuleucel-T [PAP-targeting DC vaccine] + ipilimumab [anti-CTLA-4])	IFN-γ	PBMCs from mCRPC patients (N=50; multiple timepoints pre- and post-treatment)	PAP (prostatic acid phosphatase) and PA2024 (recombinant fusion protein used in sipuleucel-T manufacture)	Not reported explicitly	Yes (IFN-γ ELISpot response to PA2024 associated with improved survival; lower pre-treatment CTLA-4+ T cells → better outcomes)	Predictive / Monitoring	Low
[100]	Zhang et al., 2022	Bladder cancer (NMIBC; orthotopic murine MB49 model +	Preclinical + Translational (orthotopic murine models +	Cancer immunotherapy (rBCG-S.FimH – engineered BCG overexpressing mannose-binding FimH protein for	IFN-γ	Mononuclear cells from tumor-draining lymph nodes	MB49 tumor-specific antigen S12 peptide + Smcy tumor-specific peptide (10	Not reported	No	Efficacy / Monitoring	Low

	First Author/ Year	Cancer Type	Study Design	Research Line	ELISPOT Readout	Cell Population	Antigen/Stimulation	Positivity Threshold	Clinical Outcome Association	Biomarker Category	ELISPOT Standardization Level
		human PBMCs)	human PBMC co-cultures)	enhanced urothelial adhesion)		(murine); 2.5×10⁴ cells/well; 48h	μg/mL); AID ELISPOT Reader				
[101]	Vardeu et al., 2022	Prostate cancer (preclinical; multiple mouse strains)	Preclinical (in vivo; C57BL/6, BALB/c, CD-1, HLA-A2 transgenic mice; IM prime + IV or IM boost)	Cancer vaccines (ChAdOx1 [IM prime] + MVA [IV or IM boost] encoding 5T4, PSA, PAP, STEAP1)	IFN-γ	Splenocytes (murine); 200,000 cells/well; 18h	PSA, PAP, 5T4, STEAP1 overlapping 15-mer peptide pools (1 μg/mL each); ConA as positive control; 0.4% DMSO as negative control	Not reported	No	Efficacy / Monitoring	Low
[102]	Liu et al., 2022	Cervical cancer (HPV+; biopsy-derived autologous cells, N=24 patients)	Preclinical + Translational (in vitro with patient samples [N=24] + murine cervical tumor U14 models)	NK cell immunotherapy + checkpoint inhibition (lirilumab [anti-KIR] + avelumab [anti-PD-L1]; Vav1-NF-κB disinhibition)	IFN-γ and Granzyme B	Blood-derived NK cells from HPV+ cervical cancer patients (N=24); co-cultured with autologous biopsy-derived	Autologous HPV+ cervical cancer cells (biopsy-derived) ± lirilumab (20 μg/mL) and/or avelumab (2.0 μg/mL); receptor crosslinking 4h	Not reported	No	Efficacy / Monitoring	Low

	First Author/Year	Cancer Type	Study Design	Research Line	ELISPOT Readout	Cell Population	Antigen/Stimulation	Positivity Threshold	Clinical Outcome Association	Biomarker Category	ELISPOT Standardization Level
						malignant squamous cells					
[103]	Schönharting et al., 2023	HER2+ metastatic breast cancer (case report; 1 patient)	Clinical (case report; bioinformatic-based personalized peptide immunization BITAP; 5 cycles)	Cancer vaccines (BITAP – BioInformatic Tumor Address Peptides; personalized neoantigens predicted from whole exome sequencing)	IFN-γ (amplified, per CIMT CIP guidelines; after 12-day in vitro stimulation + IL-2 120 U/mL)	PBMCs from 1 HER2+ metastatic breast cancer patient; tested in duplicate/triplicate	76 personalized peptides (66 class I + 10 class II; somatic mutation-derived); 12-day stimulation; ImmunoSpot Series 2.0 Analyzer	≥2-fold above negative control (medium) AND minimum 50 spots detected	Yes (stable disease with reduced tumor markers CA 15-03/CEA; improved overall survival compared to historical data)	Predictive / Monitoring	High
[104]	Tran et al., 2023	Melanoma (stage IIIB–IV; Mel51 N=9, Mel53 N=4 eligible patients)	Clinical (two Phase I/II trials: NCT00977145 [Mel51 – intratumoral IFN-γ] + NCT01264731 [Mel53 –	Cancer vaccines + intratumoral immunostimulation (12 MHC-I restricted melanoma peptide vaccine [12MP] + intratumoral IFN-γ [Mel51] or topical imiquimod [Mel53])	IFN-γ (direct ex vivo; CD8+ T cell ELISpot on cryopreserved PBMCs)	PBMCs from melanoma patients (Mel51 n=9, Mel53 n=4); multiple timepoints pre- and post-vaccination	12 MHC class I-restricted melanoma peptides (12MP) pooled	≥2× background AND ≥20 spots per 10^5 CD8+ cells above negative control	Yes (6/9 [Mel51] and 2/4 [Mel53] had circulating T-cell responses; associated with vaccine-induced tumor infiltrating lymphocytes [viTILs])	Predictive / Monitoring	High

	First Author/ Year	Cancer Type	Study Design	Research Line	ELISPOT Readout	Cell Population	Antigen/Stimulation	Positivity Threshold	Clinical Outcome Association	Biomarker Category	ELISPOT Standardization Level
			topical imiquimod TLR7])								
[105]	Pichler et al., 2023	Bladder cancer (high-risk NMIBC; N=11 patients; unvaccinated SARS-CoV-2-naïve)	Clinical / Translational (prospective longitudinal; pre-COVID-19 era samples; blood/PBMCs at baseline and during BCG treatment)	Immunomodulation / Trained immunity (intravesical BCG therapy for NMIBC; off-target innate immunity against SARS-CoV-2)	IFN-γ (SARS-CoV-2 virus-specific T cells)	PBMCs from 11 NMIBC patients (unvaccinated, SARS-CoV-2-naïve; baseline + during BCG treatment)	SARS-CoV-2 peptide pool (off-target antigen; assessing trained innate immunity)	Not reported	No (exploratory; off-target trained immunity assessment)	Monitoring	Low
[106]	Wespiser et al., 2023	Metastatic non-small cell lung cancer (NSCLC)	Clinical / Translational (dynamic monitoring study; N=12 patients receiving	Checkpoint inhibitor + chemotherapy (first-line chemo-IO; pembrolizumab-based regimens)	IFN-γ (antitumor CD4+ Th1 response)	PBMCs from 12 metastatic NSCLC patients (dynamic monitoring; multiple timepoints	Telomerase-derived tumor-associated antigens (antitumor CD4+ Th1 panel); standard recall	Not reported	Yes (expansion of systemic antitumor CD4+ Th1 response associated with clinical response to chemo-IO)	Predictive / Monitoring	Low

	First Author/ Year	Cancer Type	Study Design	Research Line	ELISPOT Readout	Cell Population	Antigen/Stimulation	Positivity Threshold	Clinical Outcome Association	Biomarker Category	ELISPOT Standardization Level
			first-line chemoimmunotherapy)			during chemo-IO)	antigen stimulation				
[107]	Barsegian et al., 2024	Metastatic castration-resistant prostate cancer (mCRPC; bone metastases)	Clinical / Translational (prospective; N=21 mCRPC patients receiving 6 cycles of radium-223; longitudinal immune monitoring)	Radionuclide therapy (radium-223 alpha emitter targeting bone metastases; immune function assessment)	IFN-γ (Th1) and IL-10 (Th2) — lymphocyte function assessment	PBMCs from 21 mCRPC patients (at baseline, months 1, 2, 4, 6 during radium-223 therapy)	Microbial recall antigens (e.g., tetanus antigen) + mitogens (to distinguish memory from naive T cell function)	Not reported	Yes (IL-10 secretion at baseline after tetanus antigen stimulation negatively correlated with tumor burden [Bone Scan Index]: p<0.0001, r=−0.82; baseline immune function predictive of 67% response rate)	Predictive / Monitoring	Low
[108]	Pandey et al., 2024	Breast cancer (4T1 murine model; in vitro BMDCs/spleen cells)	Preclinical (in vivo, 4T1 tumor-bearing BALB/c mice; in vitro bone	Immunomodulation / DC biology (PGE2 pathway: COX-2 inhibitor NS-398, EP4 antagonist MK29894, STAT3 inhibition, miR365 mimic to	TNF-α (ELISpot kit, BD Biosciences); IFN-γ and IL-6	BMDCs and splenocytes from 4T1 tumor-bearing BALB/c mice	PGE2-conditioned or COX-2/EP4/STAT3-inhibited DCs co-cultured with T cells; stimulation to	Not reported	No (preclinical mechanistic study)	Efficacy / Mechanism	Low

	First Author/ Year	Cancer Type	Study Design	Research Line	ELISPOT Readout	Cell Population	Antigen/Stimulation	Positivity Threshold	Clinical Outcome Association	Biomarker Category	ELISPOT Standardization Level
			marrow-derived DCs [BMDCs] and spleen cells)	restore DC and T cell function)	(ELISA/ ELISpot, Abcam)		assess cytokine production				
[109]	Heath et al., 2024	Metastatic castration-resistant prostate cancer (mCRPC; African American [AA, N=29] vs. non-AA [N=28] men)	Clinical (single-arm, two-cohort multicenter study; sipuleucel-T; racial differences in immune response)	Cancer vaccines (sipuleucel-T – FDA-approved PAP-targeting autologous DC vaccine)	IFN-γ (CTL activity; CD8+ and CD4+ surrogate)	PBMCs from mCRPC patients (AA n=29, non-AA n=28; 3×10⁵ cells/well; 48h at 37°C)	PA2024, PAP, PSMA, PSA (5–10 μg/mL); PHA-L as positive control; irrelevant peptide as negative control	≥5 spots per 10⁶ PBMCs; positive increase = ≥2-fold increase over baseline	No (no racial differences in IFNγ ELISpot responses against PA2024, PAP, PSA, or PSMA; exploratory)	Monitoring	High
[110]	Naik et al., 2025	Breast cancer (basal-like cell lines: MDA-MB-468, MDA-MB-231; in vitro HLA-matched co-culture)	Preclinical in vitro (siRNA knockdown of LDHC in basal-like breast cancer cell lines; direct co-	Immunomodulation / TME (LDHC knockdown as tumor-intrinsic immunomodulatory target; direct cancer cell–immune cell contact required	IFN-γ (Human IFN-γ ELISpot PLUS kit, HRP, Mabtech)	HLA-matched peripheral blood lymphocytes (PBLs); 5×10⁴ PBLs/well; after 4h direct co-culture	LDHC-silenced (siLDHC) vs. control (siCTRL) breast cancer cells as direct contact stimulators; 4h co-culture	Not reported	No (preclinical in vitro mechanistic study; LDHC knockdown significantly increased IFN-γ production across all three breast cancer cell lines)	Efficacy / Mechanism	Low

	First Author/ Year	Cancer Type	Study Design	Research Line	ELISPOT Readout	Cell Population	Antigen/Stimulation	Positivity Threshold	Clinical Outcome Association	Biomarker Category	ELISPOT Standardization Level
			culture with HLA-matched PBLs; 3 independent donors; 3 independent experiments)	for T cell activation)		with LDHC-silenced (siLDHC) cancer cells (T:E ratio 1:50)					
[111]	Muraro et al., 2022	Metastatic breast cancer (mBC; predominantly luminal subtypes; N=20 patients)	Clinical / Translational (prospective observational liquid biopsy study; combined CTCs + T-cell immunity monitoring; N=20 mBC patients	Liquid biopsy immunomonitoring (CTCs enumerated by metabolism-based DEPArray assay + IFN-γ ELISpot for antitumor T-cell immunity + TCR repertoire sequencing)	IFN-γ	PBMCs from 20 mBC patients (before therapy and 1 month after starting new therapy line)	Tumor-associated antigens (TAA) panel; standard panel for antitumor T-cell response assessment	Not reported	Yes (CTCs below cutoff combined with positive ELISpot = highest TCR clonality [p=0.03]; combined liquid biopsy biomarker associated with overall survival [p=0.03])	Predictive / Monitoring	Low

	First Author/ Year	Cancer Type	Study Design	Research Line	ELISPOT Readout	Cell Population	Antigen/Stimulation	Positivity Threshold	Clinical Outcome Association	Biomarker Category	ELISPOT Standardization Level
[112]	Liu et al., 2022	Advanced high-grade serous ovarian cancer (EOC; N=10 patients; stage III/IV)	before and 1 month after starting new therapy line) Clinical / Translational (prospective serial sampling; N=10 HGSOC patients receiving neoadjuvant carboplatin+paclitaxel [NACT]; serial PBMCs pre-NACT, after cycle 3,	Chemotherapy immunomodulation (neoadjuvant carboplatin+paclitaxel NACT; assessment of peripheral T-cell immunity evolution during treatment)	IFN-γ (ex vivo; cryopreserved PBMCs thawed and rested overnight; 18h stimulation)	PBMCs from 10 HGSOC patients (cryopreserved; serial timepoints: pre-NACT, post-cycle 3, post-cycle 6, ~2 months post-completion)	CEF peptide pool (CMV, EBV, Influenza; Mabtech) + Influenza A viral lysate (Zeptometrix); 18h; viral antigen surrogates to assess T-cell functional recovery	Not reported	No (monitoring off-target viral antigen T-cell function; improved response to viral antigens post-NACT paralleled CA125 decline; not direct antitumor response correlation)	Monitoring	Low

	First Author/ Year	Cancer Type	Study Design	Research Line	ELISPOT Readout	Cell Population	Antigen/Stimulation	Positivity Threshold	Clinical Outcome Association	Biomarker Category	ELISPOT Standardization Level
			cycle 6, ~2 months post-NACT)								
[113]	Chen et al., 2024	Lung cancer patients (clinical observational; immune responses to SARS-CoV-2 Omicron and reinfection risk)	Clinical / Observational (prospective; lung cancer patients; monitoring SARS-CoV-2 Omicron-specific T-cell immunity and reinfection outcomes)	Off-target viral immunity monitoring (SARS-CoV-2 Omicron variant immune responses and reinfection in lung cancer patients)	IFN-γ (SARS-CoV-2-specific T cells; Bioreader 4000, BIOSYS, Germany)	PBMCs from lung cancer patients (multiple timepoints; Omicron-specific T-cell quantification)	SARS-CoV-2 Omicron peptide pools (off-target antigen; monitoring viral immunity in cancer patients)	Not reported	No (exploratory; SARS-CoV-2 off-target immunity monitoring in cancer patients; no antitumor outcome correlation)	Monitoring	Low
[114]	Gangaev et al., 2024	Multiple solid cancers (lung, breast, gastrointest	Clinical / Translational (multicenter VOICE	Off-target viral immunity monitoring (mRNA-1273 COVID-19 vaccination;	IFN-γ (spike-specific T cells)	PBMCs from 386 participants (cancer patients on immunoth	SARS-CoV-2 spike peptide pools (vaccine-induced off-target immunity)	Not reported	No (spike-specific T-cell responses robust across all treatment groups [75–80%	Monitoring	Low

	First Author/ Year	Cancer Type	Study Design	Research Line	ELISPOT Readout	Cell Population	Antigen/Stimulation	Positivity Threshold	Clinical Outcome Association	Biomarker Category	ELISPOT Standardization Level
		inal, etc.; VOICE trial; N=386 participants)	trial; N=386 participants: solid cancer patients on immunotherapy, chemotherapy, or both, vs. healthy controls; mRNA-1273 COVID-19 vaccine)	spike-specific T-cell responses in cancer patients undergoing IO/chemotherapy)		erapy/chemotherapy vs. healthy controls); assessed 28 days after full mRNA-1273 vaccination			response rates]; no antitumor outcome correlation; monitoring COVID-19 vaccine immunogenicity in cancer patients)		
[115]	Li et al., 2025	Colorectal cancer (CRC; clinical + preclinical: CRC patients and C57BL/6N mouse models)	Clinical + Preclinical (in vitro co-culture with CRC patient-derived platelets; in vivo C57BL/6	Immune checkpoint inhibitor resistance (PD-L1+ platelets as immunosuppressive TME mediators via fibronectin-1/GPIbα/integrin α5β1 pathway;	IFN-γ	PBMCs (n=3) co-incubated with PD-L1+ or PD-L1− platelets isolated from CRC patients ± PD-L1	Autologous CRC patient-derived PD-L1+ or PD-L1− platelets (± anti-PD-L1 mAb pretreatment); stimulation conditions for T cell	Not reported	Yes (PD-L1+ platelets suppress T cell IFN-γ production; anti-PD-L1 pretreatment rescues T cell activity; PD-L1+ platelet status correlates with	Predictive / Mechanism	Low

	First Author/ Year	Cancer Type	Study Design	Research Line	ELISPOT Readout	Cell Population	Antigen/Stimulation	Positivity Threshold	Clinical Outcome Association	Biomarker Category	ELISPOT Standardization Level
			N murine CRC models: CD274 KO, CD8a KO, PF4-Cre-Hsp90b1 KO and aspirin + PD-L1 mAb treatment)	antiplatelet agents [aspirin] + PD-L1 mAb to overcome ICI resistance)		mAb pretreatment; expressed as Spot-Forming Units (SFU)/250,000 seeded cells minus background	activation assessment		poor prognosis and CD8+ T cell exhaustion in CRC patients)		
[116]	Dathathri et al., 2025	Prostate cancer (PCa cell lines: LNCaP, 22Rv1, PC3; CTCs from 2 castration-naïve PCa [CNPC] patients)	Preclinical in vitro (PCa cell lines representing benign, hormone-sensitive, castration-resistant stages + feasibility validatio	Secretome biomarker discovery / ADT monitoring (modified ELISpot platform adapted for single-cell PSA protein secretion; cell cycle influence on PSA secretion; anti-androgen therapy	PSA protein secretion (modified ELISpot capture assay; NOT immune cytokin	PCa cell lines (LNCaP, 22Rv1, PC3) at single-cell level; CTCs isolated from 2 CNPC patients (DLA-derived)	Anti-androgen therapy (enzalutamide/ ADT); cell cycle manipulation (FACS/cytospin synchronization); modified ELISpot antibody capture for PSA protein	Not reported (assay feasibility/methodological study)	No (preclinical feasibility; PSA secretion found only in LNCaP cells and was cell cycle-dependent; Progranulin and Cathepsin D identified as complementary secretome biomarkers in	Biomarker Discovery / Monitoring	Low

First Author/ Year	Cancer Type	Study Design	Research Line	ELISPOT Readout	Cell Population	Antigen/Stimulation	Positivity Threshold	Clinical Outcome Association	Biomarker Category	ELISPOT Standardization Level
		n with CTCs from 2 clinical PCa patients)	[enzalutamide] effects; proteome array for complementary biomarker identification)	e — adapted platform for single-cell tumor secretome analysis)	for clinical validation of assay	secretion from single PCa cells		metastatic PCa cell lines)		

* Studies showing a statistically significant association between ELISPOT responses and clinical outcomes. † Studies reporting clinical observations associated with ELISPOT responses without statistical validation. § Studies providing well-defined ELISPOT positivity thresholds, representing the best methodological practices. ‡ Studies with interpretative considerations, including classification overlaps or lack of direct correlation between ELISPOT responses and reported clinical outcomes. Note: ELISPOT standardization level was qualitatively classified based on the reporting of assay parameters, including positivity thresholds, control conditions, and reproducibility criteria. Studies were categorized as High (clear quantitative thresholds and well-defined criteria), Moderate (statistical comparisons or partial methodological definition), or Low (lack of explicit standardization criteria).
